# Integrating Structure to Protein-Protein Interaction Networks That Drive Metastasis to Brain and Lung in Breast Cancer

**DOI:** 10.1371/journal.pone.0081035

**Published:** 2013-11-22

**Authors:** H. Billur Engin, Emre Guney, Ozlem Keskin, Baldo Oliva, Attila Gursoy

**Affiliations:** 1 Center for Computational Biology and Bioinformatics and College of Engineering, Koc University, Istanbul, Turkey; 2 Structural Bioinformatics Group (GRIB), Universitat Pompeu Fabra; Aberystwyth University, United Kingdom

## Abstract

Blocking specific protein interactions can lead to human diseases. Accordingly, protein interactions and the structural knowledge on interacting surfaces of proteins (interfaces) have an important role in predicting the genotype-phenotype relationship. We have built the phenotype specific sub-networks of protein-protein interactions (PPIs) involving the relevant genes responsible for lung and brain metastasis from primary tumor in breast cancer. First, we selected the PPIs most relevant to metastasis causing genes (seed genes), by using the “guilt-by-association” principle. Then, we modeled structures of the interactions whose complex forms are not available in Protein Databank (PDB). Finally, we mapped mutations to interface structures (real and modeled), in order to spot the interactions that might be manipulated by these mutations. Functional analyses performed on these sub-networks revealed the potential relationship between immune system-infectious diseases and lung metastasis progression, but this connection was not observed significantly in the brain metastasis. Besides, structural analyses showed that some PPI interfaces in both metastasis sub-networks are originating from microbial proteins, which in turn were mostly related with cell adhesion. Cell adhesion is a key mechanism in metastasis, therefore these PPIs may be involved in similar molecular pathways that are shared by infectious disease and metastasis. Finally, by mapping the mutations and amino acid variations on the interface regions of the proteins in the metastasis sub-networks we found evidence for some mutations to be involved in the mechanisms differentiating the type of the metastasis.

## Introduction

Metastasis is the mechanism that causes the distant spread of cancer [1]. As our diagnosing and treating ability of cancer advances, the fatality is moving towards metastatic phase [2]. According to American Cancer Society, breast cancer is the second most common cause of cancer death among women [3]. The death cause of a breast cancer patient is often metastasis to an organ other than the tissue of the primer tumor. The brain, the lung and the bone are common breast cancer metastasis sites [4]. 

In the recent years, numerous studies have been trying to shed light on molecular mechanisms of metastasis. Some of them are: oncogene activation with new experimental methods [5], identifying organ specific metastasis [4,6], the identification of genes associated with metastases [1,7–10] and discovery of pathways playing role in metastasis [11]. DNA-microarray studies demonstrated that primary breast tumors developing metastasis can be distinguished from tumors that do not metastasize, using gene expression profiles [12]. Massagué and his co-workers published several papers about breast cancer metastasis in the last decade, and in particular two of them studied the metastases of breast cancer towards brain and lung. One article [4] identified18 genes that mediate breast cancer to lung metastasis, and the other [13] classified 17 genes that mediate breast cancer to brain metastasis. They used differential expression analysis to identify these genes.

Genes related with metastasis are usually biologically related with each other [1]. For this reason, analysis of individual genes does not provide solid results about the metastatic process. Network formation and analyses are important tools for systems biology, providing a powerful abstraction of intracellular complex relationships. Most common diseases such as diabetes, schizophrenia, hypertension and cancer, are also believed to be caused by multiple genes (multi-genic) [14]. Recently, genes that have the potential to be involved with several diseases are uncovered through the integration of functional information of proteins and the protein interaction network [15–17]. Interactions in the sub-networks generally indicates functional signaling cascades, metabolic pathways or molecular complexes, which gives an idea about the cause or the result of the disease (phenotype) [18]. Protein interaction networks were also used to predict genes involved in breast cancer metastasis, and to identify the disease-related sub-networks [16,19].

On the other hand, structural data can be very useful for explaining the molecular mechanisms leading to disease when used in conjunction with information about the mutation responsible for the disease [20]. For instance, Wang and colleagues [21] investigated the molecular mechanisms underlying complex genotype-phenotype relationships by integrating large-scale PPI data, mutation knowledge and atomic level three-dimensional (3D) protein structure information available in RCSB Protein Databank (PDB) [22]. They revealed that the in-frame mutations are augmented on the disease related proteins’ interaction interfaces. Similarly, David et. al. [23] combined structural data of proteins/protein-complexes and non-synonymous single nucleotide polymorphisms (nsSNPs) and they investigated the location of nsSNPs. They have observed that disease-causing nsSNPs that occur on the protein surface prefer to be located on the protein-protein interfaces. 

Integrating structure to PPI networks has recently been used to provide insights on the mechanism of interactions [24,25]. This approach may help us detect which interaction partners are competing with each other to bind the same region on a particular protein. Accordingly we can see which interactions may happen at the same time and which cannot. Knowing the structural architecture of the interactions, we may spot the protein pairs that use similar interface architectures. Given that ligands tend to bind to similar binding sites [26–28], a drug targeting any of these structurally similar PPIs will have a propensity to target the others as well [29,30]. Furthermore, identifying the interface region of two proteins enables us to discover whether mutations of these proteins happen in the interface or not. 

In order to understand the molecular mechanism of the brain/lung metastasis of breast cancer patients, we have generated lung and brain metastatic breast cancer sub-networks by finding the most relevant edges to the seed genes identified by Massagué and his co-workers. Then, we enriched these networks with structural information of 3D structural models of known protein-complexes and predicted its protein-protein interfaces. We have analyzed the protein-protein interfaces commonly employed in these sub-networks and observed that interactions of microbial origin played an important role. We also investigated the mutations happening on the most relevant proteins of the breast cancer metastasis sub-networks. Our results suggested that key protein-protein interfaces may be mediating the metastasis, in which a certain mutation could be selectively altering the interaction.

## Materials and Methods

### The Human PPI Network

Experimental data on protein interactions are spread among multiple databases. Even if the data in these databases partially overlap, the reliability of data differs because of the variations in the experimental techniques and the organisms used. In addition, information of the same protein can be stored with different designations in different databases. Therefore, all the available data should be queried properly and matches should be combined to form a comprehensive human PPI network. We made use of BIANA [31] (Biological Integration And Network Analysis) bioinformatics tool in order to form human PPI network. BIANA gathered PPI data from various databases and dealt with mapping between the different identifiers. We combined DIP[32], MIPS[33], HPRD[34], BIND[35], IntAct[36], MINT[37] and BioGRID [38] databases (all downloaded on May, 2011). Interactions and protein information were integrated with BIANA assuming that two proteins from different databases were the same if they had the same UNIPROT Accession, amino acid sequence, or Entrez Gene Identifier. 

### The Sub-Networks Implicated in Lung and Brain Metastatic Breast Cancer

We used GUILD, a network-based disease-gene prioritization tool [39] to identify the sub-networks implicated in the two phenotypes of our interest: 1) breast cancer metastasis in lung, and 2) breast cancer metastasis in brain. GUILD package includes several methods of “guilt-by-asssociation” to prioritize a list of candidate genes associated with a phenotype. Guilt-by-association approaches are based on a set of genes associated with a phenotype, named seeds, and the tendency that other genes associated with the same phenotype will interact with the seeds. We took 18 genes that mediate breast cancer to lung metastasis [4], 17 genes mediating breast cancer to brain metastasis [13] identified by Massagué and his co-workers and used them as seeds for each phenotype (Table **1**). 

**Table 1:** Metastasis seed genes.

**LUNG METASTASIS SEEDS**	**BRAIN METASTASIS SEEDS**
MMP1*	MMP1*
RARRES3	RARRES3
FSCN1*	FSCN1*
ANGPTL4	ANGPTL4
LTBP1	LTBP1
PTGS2	PTGS2
KYNU	SEPP1
TNC	LAMA4*
C10orf116	PLOD2*
CXCL1	COL13A1
CXCR4*	SCNN1A*
KRTHB1* (KRT81)	RGC32
VCAM1	PELI1
LY6E	TNFSF10*
EREG	B4GALT6
NEDD9*	HBEGF*
MAN1A1	CSF3
ID1*	

18 genes [4] that mediate breast cancer to lung metastasis, and 17 genes [13] that mediates breast cancer to brain metastasis. (*) Implies the genes, whose protein products are hubs in the metastasis sub-networks.

We employed the NetCombo algorithm in GUILD using the default parameters as in [39] to rank all the proteins of the major component of the human PPI network. This algorithm combines the algorithms of NetScore, NetZcore and NetShort. The scores were different for proteins produced by genes associated with brain metastasis than those associated with lung metastasis. Therefore, two different sub-networks were considered with the proteins associated with lung or brain metastasis and their interactions. 

GUILD scored only the nodes (proteins/genes) but not the edges (PPIs) and gene-gene associations), therefore we needed to transfer the score of the nodes into the edges. Thus, we defined the score of the edge as the average of the scores of its nodes (the values of these scores lie between 0 and 1). We selected a common threshold cut-off on the score of the edges to set up the sub-networks of brain and lung metastasis with similar size.

### Gene Expression in the Related Tissues

We used HPRD [34], UNIPROT [40,41] and TIGER[42] databases for checking the expression of genes in breast tissue.

### Functional Analysis of Brain and Lung Metastatic Networks

We used the ClueGo [43], a Cytoscape [44] plugin, designed for biological interpretation of gene sets. The significance (enrichment) analysis was performed with right-sided hyper-geometric testing with a Bonferroni step down P-value correction factor. KEGG pathways used for the calculations are downloaded in 24.05.2012. P-values smaller than 0.05 were considered significant.

### Introducing Structural Information to Metastasis Sub-Networks

We used PRISM [45–47] server, for predicting the interface structures of interacting protein-protein couples. An interface is the contact region between two interacting proteins. In our study interfaces consist of PDB chains. Interface templates are the available structures of protein complexes. They are the PDB structures of interacting proteins. PRISM bioinformatics tool predicts possible interactions, and how the interaction partners connect structurally, based on geometrical comparisons of the template structures and the target structures. The details of this method can be found in a previous publication [47].

An interface template consists of two chains of a PDB structures. The template is named with the combination of PDB ID and chain names. For example, in Figure **S1** in File **S1** FN 1 and LTBP1 are predicted to be interacting via 1ywkAC template. This template is originating from A and C chains of structure with the 1ywk PDB ID.

In recent years, we applied PRISM algorithm on various signaling pathways and obtained reasonable structural models of the unknown interactions [29,48,49]. The performance of the PRISM algorithm was recently investigated on standard docking benchmarks and was established to be comparable to other rigid docking approaches, though, noticeably more efficient [50]. 

PRISM uses template based prediction approach, and needs the 3D structure of the queried proteins. It cannot make estimation for a protein, which does not have a 3D structure deposited to PDB. Accordingly if an edge is not connecting two proteins whose 3D structures are available, PRISM will not be able to find results for that edge. Thus, via the PDB we searched for the 3D structural information of the proteins of lung metastasis sub-networks (LMSN) and brain metastasis sub-networks (BMSN). Brain metastasis network has 255 proteins and for 117 of them we found 1612 PDB structures. On the other hand, LMSN has 322 proteins and for 182 proteins we found 2712 PDB structures. In BMSN there are 58 interactions connecting proteins with known structure stored in the PDB (these interactions can be modeled with PRISM) and in LMSN there are 102 such interactions. This means that, we could only make models for these edges.

We eliminated redundancy of similar structures corresponding to the same interface using TM-align[51]. Accordingly, we grouped PDBs that have a TM-score greater than 0.5 and an RMSD score smaller than 2.5A. We chose one representative for each group of PDBs that describe the same region. We ended up with 255 PDB structures for 117 proteins of the BMSN, and with 414 PDB structures for 182 proteins of the LMSN. 

In this experiment we have used 7922 interface templates (mined in 2006 from PDB) [47]. We filtered the PRISM results by considering only the interaction predictions with an energy value lower than 0. For each interface model PRISM structurally compares 2 PDB chains (target chains) to all 7922 interface templates. PRISM made multiple predictions for some of the interactions; we used the models with the lowest free binding energies.

### Source Organisms of the Templates

We have used “uniprot_sprot.dat” (downloaded in November of 2012, from UNIPROT’s ftp server) for detecting the source organisms of the PDB chains used for modeling the protein complexes in both metastasis networks.

For significance testing, we have calculated the p-value of a hyper-geometric distribution using the R package[52]. P-values smaller than 0.05 were considered significant. Please refer to Table **2** for the numbers we have used for calculations.

**Table 2:** The table for the source organism distribution of template chains, used for modeling the complexes of BMSN and LMSN.

	LMSN Template Chains	BMSN Template Chains	All Template Chains in the Dataset
Eukaryota	60	22	5822
Archaea	12	4	515
Viruses	6	4	716
Bacteria	72	26	4202
Microbial (Viruses+Bacteria)	78	30	4918
Total Number of Template Chains	150	56	11255

Every protein-protein interface consists of two chains. The 7922 template interfaces used in the experiments, consist of 15844 template chains. Among them the source organism of 11255 were available in “uniprot_sprot.dat” and 4918 were coming from microorganisms (bacteria/virus).

### Interface Clusters

The protein interfaces that are available in PDB are clustered according to their structural similarity. These clusters are provided in PRINT database which can be accessed from the http://prism.ccbb.ku.edu.tr/interface/ address. We mentioned these structurally similar protein clusters as PRINT clusters all through the text.

While detecting the source organisms of the template interfaces, we have taken into account all the interfaces, not only the representative interfaces (in each PRINT cluster). Besides, we have used the biological process and the molecular functions listed in UNIPROT database for our analyses.

### Host-Pathogen Relationships

We have made use of UNIPROT and HPIDB[53] databases to mine the knowledge on the host-pathogen relationships of the related proteins. We have checked whether the proteins are known to be interacting with pathogens or not Table **S1** in File **S1** and Table **S2** in File **S1**).

### Determining Hub Proteins

The average node degree is 2.6 for BMSN and 2 for LMSN. Nodes with 12 or more edges are considered to be hubs.

### Genetic Variations on Interface Surfaces

We obtained the available point mutations related with cancer from COSMIC [54] database and humsavar.txt of UNIPROT database. UNIPROT [40,41] provides the variants of a protein’s amino-acid sequence. These variations can be polymorphisms, variations between strains, isolates or cultivars, disease-associated mutations or RNA editing events. Both databases provide detailed information about the mutations, as well as the mutated residue numbers. Then, we mapped these point mutations to the interface regions of interacting proteins in the metastasis sub-networks (BMSN and LMSN). We used the PDBSWS database [55] for the PDB and Uniprot residue-level alignment. 

We used Naccess [56] for determining the surface and core residues. Naccess computes the atomic accessible area by rolling a probe (typically with the same radius as water (1.4 Angstroms)) around the Van der Waal’s surface of macromolecule. It employs the Lee & Richards method [57], whereby a probe of given radius is rolled around the surface of the molecule, and the path traced out by its centre is the accessible surface.

 For the statistical calculations of location preferences of genetic variations we used fisher’s (exact) test and two-tailed P-value for statistical significance (P-value smaller than 0.05 was considered statistically significant) as described in David et al.’s [23] article. We used the R package[52] for the statistical calculations.

### Hot Spot Prediction

Hot spots are the residues that contribute more to the binding free energy with respect to other residues in the protein-protein interface. We have used HotPoint [58] for hot spot predictions. This webserver calculates the hot spots in protein interfaces using an empirical model with 70% accuracy. 

### Visualization Tools Used For Figures

We have used VMD [59] for visualizing protein structures and for network visualizations we have used Cytoscape [44].

## Results and Discussion

### Identifying Brain & Lung Metastatic Breast Cancer Sub-networks and Their Functional Annotations

We have built a comprehensive human PPI network that consisted of 11,123 proteins and 149,931 interactions. We ranked each PPI in the network, according to its relevance to the seed nodes causing breast cancer metastasis, using GUILD (Genes Underlying Inheritance Linked Disorders) network-based prioritization tool [39].

We defined a score threshold and discarded interactions below the threshold based on the following reasoning: 1) we need two comparable sets of nodes and edges for brain and lung metastasis, where the topology may be different but not the size; 2) predicting the interface structures of interacting proteins is a highly time-consuming step, therefore we needed to reduce the network to a limited sub-network of small but highly relevant edges (i.e. less than 500) for each metastasis under study.

**Figure 1:**
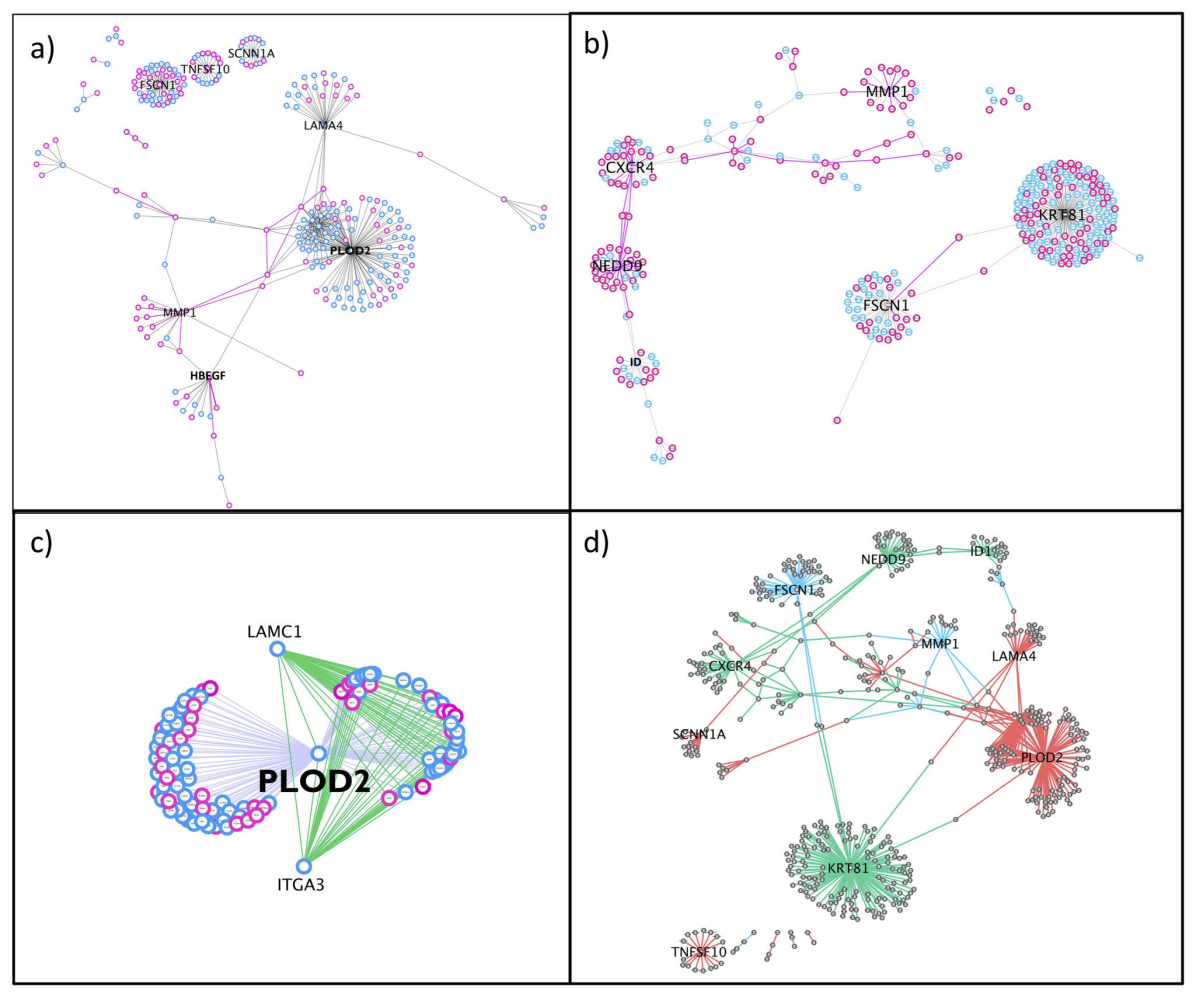
The BMSN and the LMSN networks. We obtained a) the BMSN and b) the LMSN by choosing the edges of human PPI network with GUILD Score higher than 0.178. The proteins that have PDB structures are highlighted in pink, plus the edges that have complexes modeled by PRISM are also in pink color. c) PLOD2 cluster (the first-degree neighbors of PLOD2) from the BMSN d) BMSN and LMSN merged as a one big network. There are 84 common proteins and 71 common PPIs (blue edges). The edges that are only present in LMSN are shown with green and the edges that are only present in BMSN are shown with pink.

We plotted the number of edges versus their scores to select the best cut-off (see Figure **S2** in File **S1**). We observed a dramatic rise in the number of interactions (and also nodes), between scores 0.15 and 0.18 for the punctuation of brain and lung metastasis (**Figure S3 **in File **S1** **and Table 3**). Accordingly, we selected 0.178 as the common GUILD cut-off score to generate both sub-networks. This cutoff yielded a BMSN with 255 nodes and 335 edges (Figure **1a**), and a LMSN with 322 nodes and 327 edges (Figure **1b**). 

**Table 3:** The number of edges and nodes of metastasis networks according to Guild Scores.

	BRAIN METASTASIS		LUNG METASTASIS	
CUTOFF VALUES	**#OF NODES**	**#OF EDGES**	**#OF NODES**	**#OF EDGES**
Score 0.140	276	5382	354	7085
Score 0.170	255	4220	322	328
Score 0.178	255	335	322	327

Although we used all proteins of both sub-networks (BMSN and LMSN) in our analyses, we tracked down the evidence for the expressions of the genes that coded the proteins in both sub-networks in breast tissue. We found that 87% of the genes in the LMSN (280 out of 322, see Table **S3** in File **S1**) and 93% in the BMSN (238 out of 255, see Table **S4** in File **S1**) are expressed in breast tissue. 

**Figure 2:**
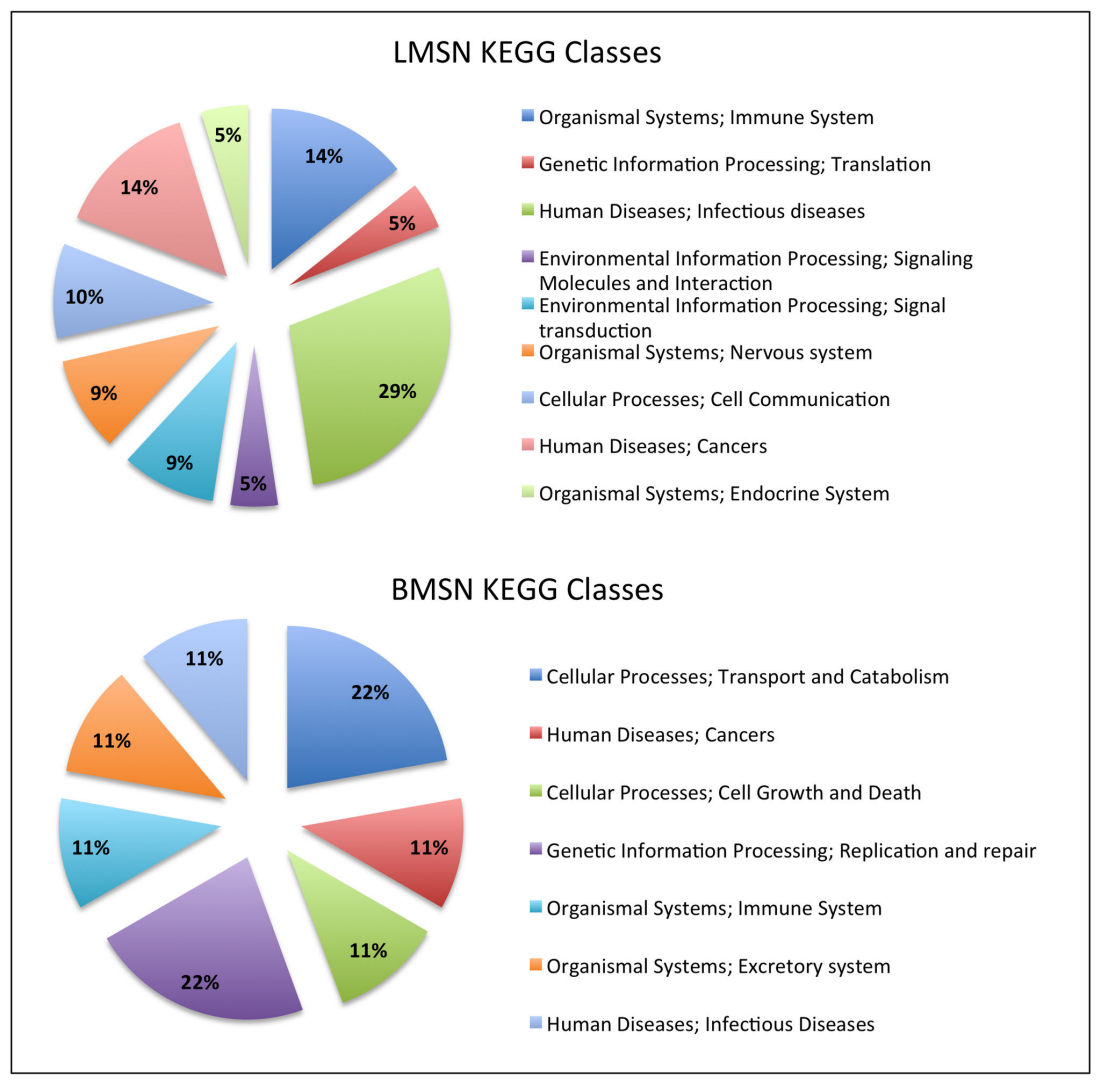
The percentages of KEGG classes observed in LMSN and BMSN.

We used ClueGo [43] to find significant KEGG pathways in BMSN (**Table S5 **in File **S1**) and LMSN (**Table S6 **in File **S1**). Each pathway in KEGG belongs to a class according to KEGG Orthology (KO) [60]. Then we mapped each KEGG pathway to its KEGG class. Subsequently, we calculated the percentages of observed KEGG classes (Figure **2**). We found out that “Transport and Catabolism Cellular Processes” and “Replication and Repair Genetic Information Processing” classes contain the most abundant significant pathways in BMSN “Infectious Diseases”, “Cancer” and “Immune System” were the classes of most abundant pathways in the LMSN.

According to the functional analysis we have observed a functional link between lung metastasis of breast cancer, infectious diseases and immune system. Although, BMSN was also significantly enriched in some pathways that are governed by “Immune system” and “Infectious Diseases”, these two classes were not covering the most abundant pathways. It is interesting that immune system and infectious diseases seem to play an important role in lung metastasis, while transport and catabolism seem to play a major role for brain metastasis. Indeed, lung tissue is in contact with the environment, being likely prepared for infection, while brain is separated of circulating blood by the blood-brain barrier and it requires metabolic processes to transport and catabolize glucose. Still, these results are obtained for networks which expression is produced mostly in breast.

### Structural Analysis of the Metastasis Sub-Networks

The network representation of PPIs provides information about the sets of interacting proteins (i.e. whether two proteins bind or do not bind and the number of interactions a protein can have). Introducing structural knowledge to PPI networks adds an extra dimension of data to the representation. When we know how proteins are interacting structurally, we can detect multiple proteins trying to bind the same region on a protein surface. This extra knowledge may help us realize which interactions cannot happen concurrently. Besides, there may be protein pairs interacting via similar interface architectures. A drug targeting on any of these PPIs will have a high probability of targeting the others as well [29,30], since ligands have tendency to bind to similar binding sites [26–28]. Moreover, knowing the interface region of two proteins helps us to check whether mutations of these proteins occur in the interface or not.

Among the PPIs of the BMSN, only 4 of them had 3D structural data of the binary complex in PDB. Similarly, for LMSN, only 2 PPIs were found with the structure of the binary complex in PDB (see Table **4**). In order to increase the structural coverage of interactions of our sub-networks, it is necessary to use modeling. We used PRISM [45–47] in order to predict, assign and model the structure of the interface of protein-pairs in the BMSN and LMSN (see Methods for the details). 

**Table 4:** Interactions available in PDB.

**Metastasis Network**	**PROTEIN NAME**	**PROTEIN NAME**	**PDB ID for THE COMPLEX**
BMSN	TNFRSF10B	TNFSF10	1D0G, 1D4V, 1DU3
BMSN	ITGA5	ITGB1	3VI4, 3VI3
BMSN	MMP1	TIMP1	2J0T
BMSN	CSF3	CSF3R	2D9Q
LMSN	MMP1	TIMP1	2J0T
LMSN	CXCL12	CXCR4	2K03, 2K04, 2K05

In PDB 4 of the PPIs of brain metastasis network had 3D structural data in their complex forms. Similarly, only 2 were found for lung metastasis network.

PRISM produces template-based predictions and it models the structure of an interaction based on the known 3D structure of two interacting proteins. The BMSN has 58 interactions with known 3D structures for both partners. LMSN has 102 such interactions. PRISM modeled 18 out of 58 interactions as a binary complex in the BMSN (see Figure **1a**). For the LMSN, 50 out of 102 interactions were modeled (see Figure **1b** and Table **5**). 

**Table 5:** Edges in both metastasis sub-networks.

	**BRAIN**	**LUNG**
**Number of Edges**	335	327
**Edges that may be Modeled**	58	102
**Edges Modeled**	18	50

BMSN has 335 edges, among which 58 are connecting two proteins with 3D structures. Thus, only 58 of them may be modeled by PRISM. PRISM predicted 18 of them. Besides, LMSN has 327 interactions. Among them, 102 are connecting two proteins that have 3D structures. PRISM preformed predictions for 50 of those 102 edges.

We should note that PRISM can model an interaction using structurally different interface templates or can use the same template interface to model different interacting protein pairs. Besides, a protein may be embodied with different chains (as identified in the PDB) or domains describing different portions or protein-states (i.e. due to post-transcriptional modifications). Therefore, the interaction between two proteins can imply more than one interface region (i.e. produced by two or more pairs of domains) that may or may not occur at the same time. This would explain the causes for multiple interface predictions. On the other hand, template interfaces can be assigned to several interactions, some of them being common for different sub-networks or highly frequent in some sub-network. This arises a particular interest because it can explain a phenotype but also has implications on the putative use of drugs disrupting a particular set of interactions. As a consequence, for BMSN we obtained 32 predictions for 18 PPIs coming from 28 interface templates. Therefore, the average template interface frequency in BMSN is 1.14 (32/28). For LMSN, we obtained 99 predictions for 50 interactions and 75 out of 99 corresponded to different template interfaces. Thus, the average template interface frequency for LMSN is 1.32 (99/75). The numbers of occurrences of interfaces in both metastasis networks are shown in Table **S7** in File **S1**. 

**Figure 3:**
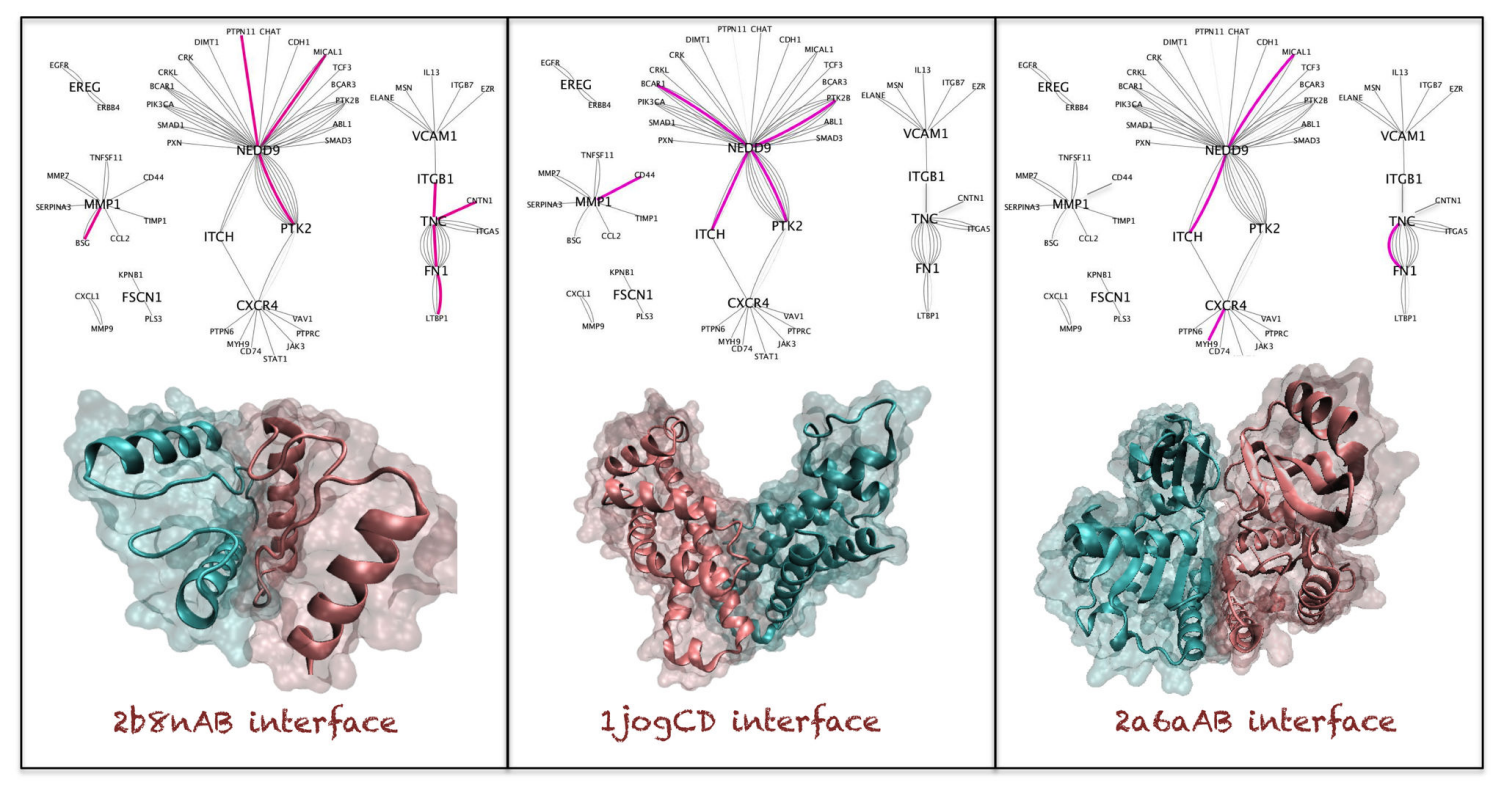
Commonly observed interfaces of lung metastasis network. In this figure structural sub-networks are also included. In these sub-networks only the interactions that have PRISM modeled complex structures are present. Each node represents a protein that has 3D structure and each edge stands for a distinct model between two proteins. The relevant template interfaces are represented with pink edges in these structural sub-networks.

**Figure 4:**
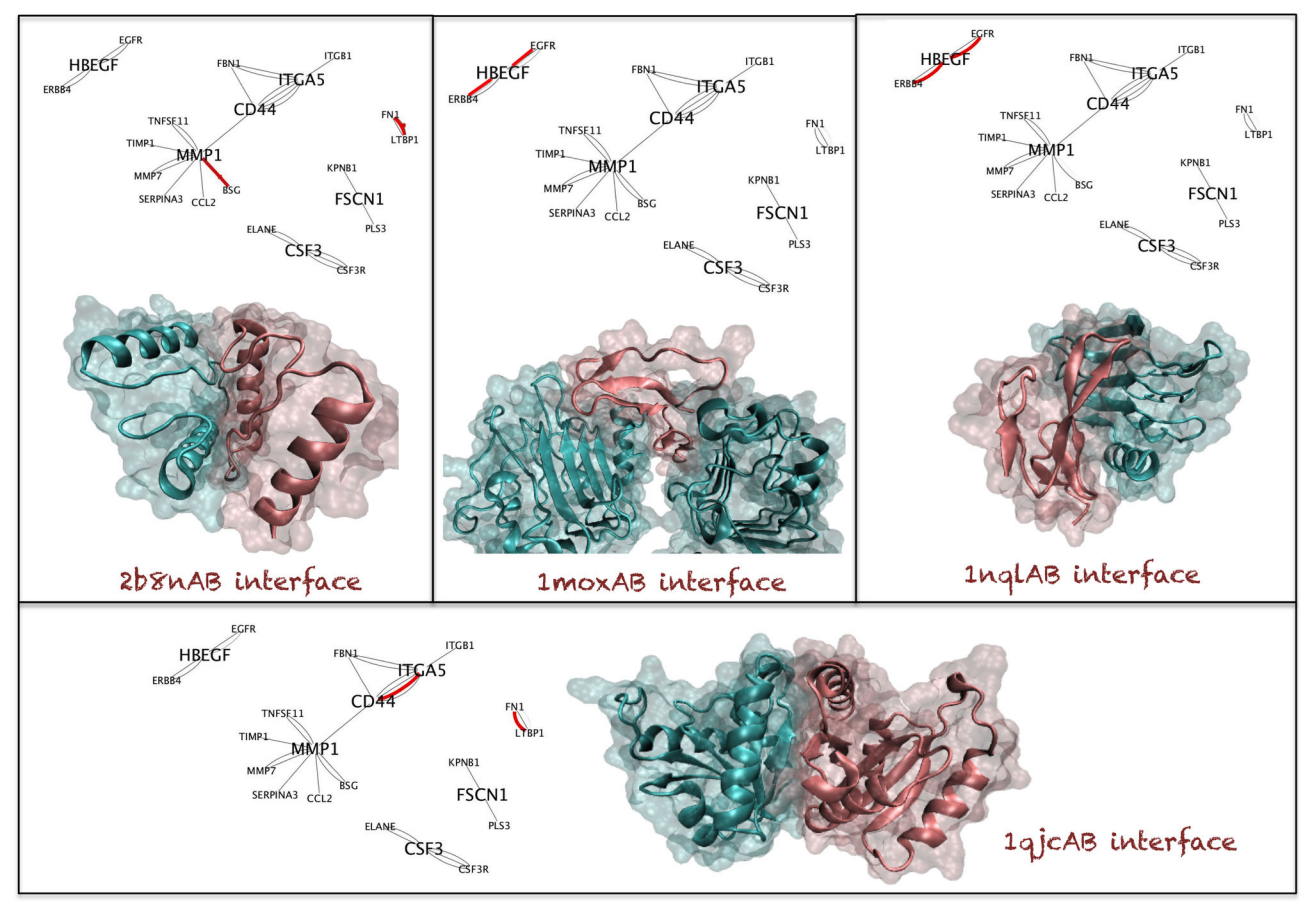
Commonly observed interfaces of brain metastasis network. Legend for the sub-networks is the same as in Figure **3**.

We studied the common template interfaces in the BMSN and LMSN. We observed top 3 high frequency template interfaces in the LMSN: 1) 2b8nAB 8 times, the interface extracted from the homodimer Glycerate kinase, putative. 2) 1jogCD 5 times, the interface extracted from the homodimer Uncharacterized protein HI_0074. 3) 2a6aAB 4 times, the interface extracted from the homodimer Peptidase M22 glycoprotease. We observed 4 template interfaces with less frequency (only in 2 PPIs) in the BMSN: 1) 2b8nAB (as for LMSN), 2) 1nqlAB, taken from the interface between EGFR-EGF, 3) 1qjcAB the interface extracted from the homodimer phosphopantetheine adenylyltransferase and 1moxAC (the interface between EGFR-TGFA). Interestingly, the 2b8nAB template interface is the most frequent interface in both sub-networks (see Figure **3** and Figure **4**). Details of the most frequent interface templates can be found in Table **6** and Table **7**. We observed that the three most common interface templates in LMSN are all coming from bacterial proteins. 

**Table 6:** Most frequently used interfaces while modeling the interactions of lung metastasis network.

**Template Interface**	2b8nAB	1jogCD	2a6aAB
**Proteins of the Template Interface**	Glycerate kinase, putative	Uncharacterized protein HI_0074	Peptidase M22 glycoprotease
**PRINT Cluster Size**	1	17	1
**# of PPIs Modelled**	8	5	4
**# of Proteins Using This Interface**	11	7	7
**Source Organism**	Thermotoga Maritima bacteria	Eukaryote and Bacteria (**Table S16 **in File **S1**)	Thermotoga Maritima bacteria
**Common Biological Processes (of Proteins of Template Interface)**	N/A	oxygen transportation (**Table S16 **in File **S1**)	hydrolase and protease
**Common Molecular Functions (of Proteins of Template Interface)**	enzymatic activities like kinase, oxidoreductase, transferase	N/A	N/A
**Common Biological Processes (of Proteins Using This Interface)**	cell adhesion (**Table S17 **in File **S1**)	cell adhesion, angiogenesis, host-virus interaction, immunity (**Table S17 **in File **S1**)	cell adhesion, cell shape and host-virus interaction (**Table S17 **in File **S1**)
**Common Molecular Functions (of Proteins Using This Interface)**	enzymatic activities (**Table S18 **in File **S1**).	enzymatic activities (**Table S18 **in File **S1**)	N/A (**Table S18 **in File **S1**)

**Table 7:** Most frequently used interfaces while modeling the interactions of brain metastasis network.

**Template Interface**	2b8nAB	1qjcAB	1nqlAB	1moxAC
**Proteins of the Template Interface**	Glycerate kinase, putative	coaD	EGFR-EGF	EGFR-TGFA
**PRINT Cluster Size**	1	7	1	4
**# of PPIs Modelled**	2	2	2	2
**# of Proteins Using This Interface**	4	4	3	3
**Source Organism**	Thermotoga Maritima Bacteria	E. Coli and Thermatoga Maritime Bacteria	Homo Sapiens	Homo Sapiens
**Common Biological Processes (of Proteins of Template Interface)**	N/A	Coenzyme A biosynthesis	N/A	N/A
**Common Molecular Functions (of Proteins of Template Interface)**	enzymatic activities like kinase, oxidoreductase, transferase	nucleotidyltransferase and transferase	Developmental Protein, Kinase, Receptor, Transferase, Tyrosine-protein kinase, Growth Factor	Developmental Protein, Kinase, Receptor, Transferase, Tyrosine-protein kinase, Growth Factor, Mitogen
**Common Biological Processes (of Proteins Using This Interface)**	N/A (**Table S19 **in File **S1**)	Cell adhesion (**Table S19 **in File **S1**)	Apoptosis, Lactation, Transcription, Transcription, Regulation (**Table S19 **in File **S1**)	Apoptosis, Lactation, Transcription, Transcription Regulation (**Table S19 **in File **S1**)
**Common Molecular Functions (of Proteins Using This Interface)**	N/A (**Table S20 **in File **S1**)	Receptor (**Table S20 **in File **S1**)	Developmental Protein, Kinase, Receptor, Transferase, Tyrosine-protein kinase, Growth Factor , Activator (**Table S20 **in File **S1**)	Developmental Protein, Kinase, Receptor, Transferase, Tyrosine-protein kinase, Growth Factor, Activator (**Table S20 **in File **S1**)

Then we studied the source organisms of all the template interfaces used in our sub-networks. We used 28 different template interfaces (**Table S7 **in File **S1**) for modeling the complexes in BMSN. Each template interface consists of 2 chains, thus there are 56 template interface chains utilized for the predictions. Among them, 30 template interface chains are originating from microbes (bacteria/virus). The probability of observing 30 or more microbial chains in a randomly selected set of 56 template interface chains is not significant (p-value = 0.09). Likewise, there were 150 template interface chains (75 template interfaces see Table **S7** in File **S1**) used for the modeling of LMSN’s complexes. 78 out of 150 template interface chains are coming from microbes. Observing 78 or more template interface chains found in microbes in a randomly selected set of 150 is significant (p-value=0.024). Thus, metastasis protein complexes may be mimicking microbial interface architectures to form complexes, although only for LMSN this feature is significant. 

**Figure 5:**
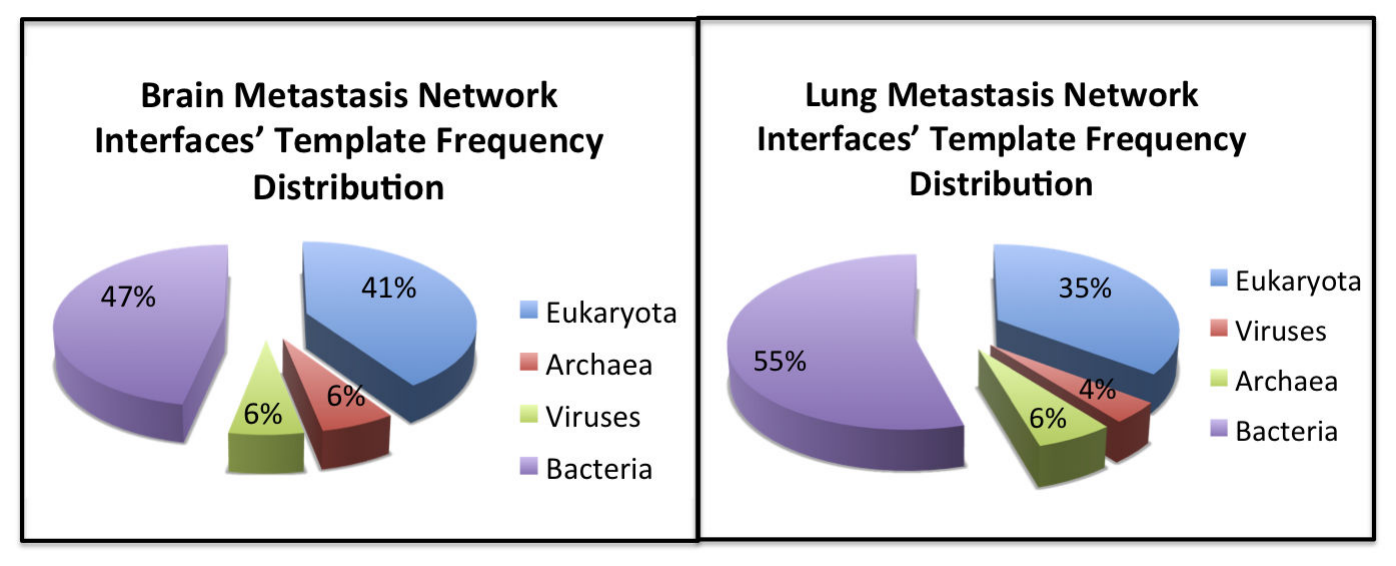
Percentages of source organisms. We considered the interfaces’ number of observations in the networks. 53% of the modeled complexes use microbial template interfaces in BMSN and this percentage is 59% in LMSN.

Then we investigated the interactions modeled with templates of protein interactions found in microbes. 53% of the models are coming from microbial origin in BMSN (Figure **5** **, left**) and 59% of the models are coming from microbes in LMSN (Figure **5** **, right**). Again, the protein complexes in LMSN, utilize more interface templates with microbial origin than the ones in brain network.

There are 14 proteins in BMSN whose interactions are modeled via templates originating from microbes. Seven out of these 14 proteins (**Table S1 **in File **S1**) are actually known to be involved in host-pathogen interactions. For LMSN this ratio is 14/40 (**Table S2 **in File **S1**). These proteins have binding sites similar to microbial interfaces and some of them are observed to be involved in the host-pathogen protein-protein interactions This finding suggests that these metastasis related proteins might be involved in mechanisms shared by metastasis and infectious diseases.

Likewise, except 1nqlAB and 1moxAC templates, all the common interfaces observed in both metastasis sub-networks are coming from bacteria. The human proteins in our networks, which are using these frequent templates, have mostly cell adhesion biological process. Moreover, 50 % of all the proteins modeled with microbial templates in our sub-networks are related with cell adhesion (**Table S8 **in File **S1** **– Table S9 in File S1**). Besides, in BMSN, 25% of the proteins modeled with non-microbial interface predictions are related with cell adhesion. Finally, 21% of the proteins in the LMSN use non-microbial interface architecture (an interface other than microbial interfaces) to interact. Cell adhesion molecules play a significant role in cancer metastasis [61,62]. Those molecules use mechanisms of cell adhesion for creating metastasis in another organ [63]. Proteins using bacterial interface architectures for interacting with other proteins may be reproducing the adhesion ability of the bacterial proteins.

Moreover, both functional analysis discussed above and the structural analysis suggest a relationship between pathogens, immune system and metastasis. Pathogens may be triggering some mechanisms that lead to metastasis of a primary breast cancer tumor or vice-versa, metastasis may create the proper environment for bacteria invasion.

Actually, previous studies highlighted the resemblances in cellular and molecular mechanisms of invasion between metastasis and infectious diseases [64–67]. Besides, in a recent study, Haile et al. hypothesized that metastasis process and pathogens should be utilizing the same pathways [68]. Liu et al. also mentioned that certain pathogens, activated immune cells and tumor cells may be sharing same tactics to spread in the body [69]. These findings reinforce our functional and structural analyses results.

### Overview of the Lung/Brain Metastasis Sub-networks

Network representation of the proteins and their interactions provides a systems level abstraction. Via network representation we may identify the proteins that are central and important. Hubs, proteins with a high number of interactions, are the vulnerable points of scale-free networks and are very important. As expected the hub proteins in the LMSB and BMSN are actually the protein products of the seed genes mentioned earlier. However, not all of the seed genes’ products are hubs in these two networks (Table 1). In BMSN PLOD2, HBEGF, MMP1, LAMA4, FSCN1, TNFSF10 and SCNN1A are the hubs (Figure **1a**), whereas in LMSN KRT81 (KRTHB1), FSCN1, ID1, NEDD9, CXCR4, VCAM1 and MMP1 are the hubs (Figure **1b**). Consequently, these seed genes are more critical from a systems point of view. 

Furthermore, there are 2 hub nodes, LAMC1 and ITGA3, in BMSN that are not seed genes. They became hub nodes in the network because of the their interactions with PLOD2’s interaction partners (shown with green edges in Figure **1c**). PLOD2 cluster (the first degree neighbors of PLOD2) is shown in Figure **1c**. They have a very high potential of being major players in brain metastasis formation. In fact, ITGA3 is down regulated in metastatic medulloblastoma tumors and claimed to be allowing metastatic tumors to spread more eagerly [70]. 

There are 84 common proteins and 71 common PPIs (blue edges in Figure **1d**) in both metastasis networks. There are PPIS present only in LMSN (green edges in Figure **1d**) and only in BMSN (pink edges in Figure **1d**). As one can see from Figure **1d**, FSCN1 and MMP1 are two hubs that are common to both metastasis sub-networks, thus they are not very helpful in differentiating two metastasis types. On the other hand, the interactions of PLOD2, the highest ranked protein in BMSN, are only present in BMSN. Similarly, KRT81 is the highest ranked protein in LMSN and its interactions are only present in LMSN. These two proteins may be playing key roles in the related metastasis types.

In Figure **1a** and Figure **1b** the proteins that have PDB structures are shown with pink nodes, while the proteins that don’t have PDB structures are shown with blue nodes. Most of the hub nodes do not have PDB structures, thus we couldn’t make further structural analyses for them. The edges that are modeled with PRISM are shown in pink in Figure **1a** and Figure **1b**. 

In Figure **3** and Figure **4** the most frequently observed template interfaces in LMSN and BMSN are depicted. In these figures structural sub-networks are also included. In these sub-networks only the interactions that have PRISM modeled complex structures are present. Each node represents a protein that has 3D structure and each edge stands for a distinct model between two proteins. The relevant template interfaces are represented with pink edges in these structural sub-networks. According to structural sub-network of lung metastasis NEDD9 is a hub protein with multiple interface architectures on different regions of its surface. 

### Genetic Variations on Interface Surfaces

There are 6 proteins that are present in both metastasis sub-networks and have at least one different interaction partner in each network. We wanted to find out whether the reason why these proteins are changing partners is related with genetic variations. By mapping the mutations on the proteins’ 3D structure we may see if the mutation is on the interface region and if the mutated residue is a hotspot, which may intensely affect the interaction strength. 

We have PRISM models for 12 interactions that these 6 proteins are involved in (Table **8**). These 12 interactions are happening between 13 proteins. By using the genetic variation data in UNIPROT and COSMIC we made further investigations for them. There are 386 genetic variations taking place on the mentioned 13 proteins; 251 variations on the surface, 135 variations in the core. Among these 386 genetic variations, only 28 of them are happening on the interface regions. Even in recent publications it is mentioned that in-frame mutations [21] and disease causing SNPs [23] have a tendency to occur on protein-protein interfaces we have not encountered this phenomenon (**Table S10 **in File **S1** **and Table S11 **in File **S1**). However, if we had a larger protein set, this result might have been different. Plus the structural information we have on interfaces is very limited, most probably we are missing some additional interfaces. Thus the genetic variations mapped on the surface region may be coinciding with interfaces as well. 

**Table 8:** List of proteins that exist in both metastasis network and the different interactions they make in each metastasis network.

**PROTEIN**	**BRAIN NETWORK INTERACTION PARTNERS**	**LUNG NETWORK INTERACTION PARTNERS**
ELANE	CSF3	VCAM1
EGFR	HBEGF	EREG
ITGA5	ITGB1 CD44 FBN1	TNC
ERBB4	HBEGF	EREG
CD44	FBN1 ITGA5 MMP1	MMP1
FN 1	-	TNC

Two of the interactions that have genetic variations on their interface regions are discussed further as case studies below.

### EGFR and ERBB4

**Figure 6:**
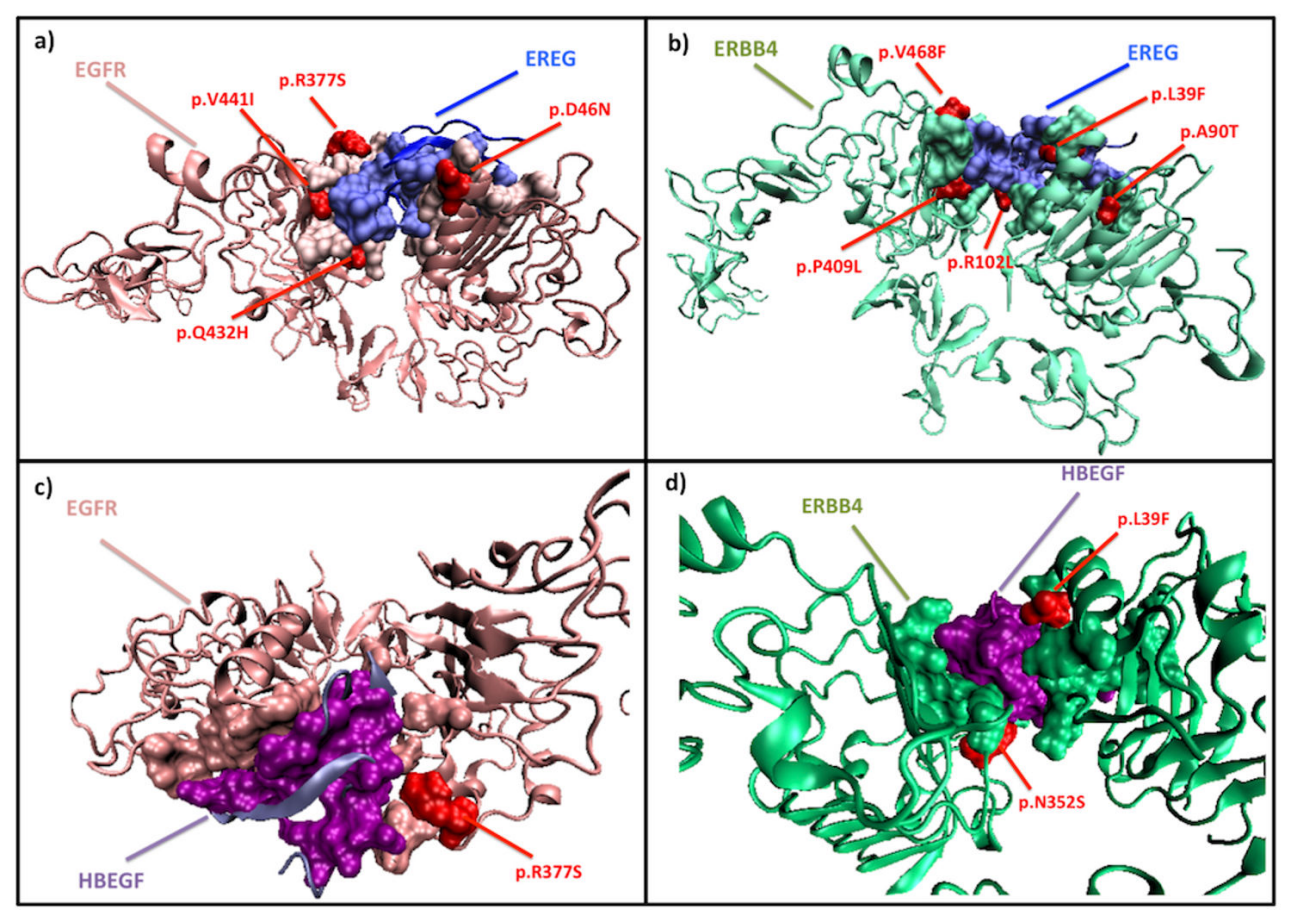
The PRISM predictions for a) EREG (blue) – EGFR (pink), b) EREG (blue) – ERBB4 (green) interaction, c) HBEGF (purple) - EGFR (pink) interacrion and d) HBEGF (purple) – ERBB4 (green) interaction. We have discovered multiple genetic variations happening on these interfaces.

The EGFR and ERBB4 proteins interact with HBEGF in BMSN, whereas they interact with EREG in LMSN. In fact HBEGF is a gene known to have a role in brain metastasis of breast cancer [13], while EREG is a gene known to be mediating lung metastasis of breast cancer [4]. The structural models of these interactions are not available in PDB, but we have PRISM predictions for these complexes. HBEGF is predicted to interact with EGFR and ERBB4 via the same binding site on its surface, and this is also the case for EREG (Figure **6**). 

Both EREG and HBEGF are growth factors that may be integrated to the membrane and can also be present in the extracellular space. EGFR binds EGF family members via its L1 (between residues 1-151) and L2 (between residues 312-481) domains [71]. The interface residues modeled with PRISM on EGFR (interfaces with HBEGF and EREG) are lying in these domains. Similar to EGFR; ERBB4 binds to EGF family members via its L1 and L2 domains (between residues 27-198 and 324-517 [72]). Most of the interface residues of ERBB4 modeled by PRISM are coinciding with these domains as well. Plus, the EGF-like domain (between residues 20-208) of HBEGF is known to have an important role in binding to EGFR [73]. The predicted interface residues for HBEGF are taking place in its EGF-like domain. EREG’s C-terminal (between residues 96-106) is suggested to be involved with its binding to ErbB receptors[74]. The C-terminus of EREG is in the interface model produced by PRISM.

There are a number of EGFR complexes, one ERBB4 complex and one HBEGF complex available in PDB, while there are no EREG complexes. When we compare our model’s interface residues with the binding sites of the available PDB complexes, we see that they are all overlapping (see Table **S12** in File **S1**, Table **S13** in File **S1**, Table **S14** in File **S1** and Table **S15** in File **S1**).

Position 102 in the amino-acid sequence of EREG acquires a SNP (p.R102L) in some cancer patients (derived from COSMIC database). This amino acid is on the interface region of EREG-ERBB4 interaction. Plus, this residue lies in the C-terminal of EREG that is known to be essential for its interactions with ErbB receptors. Moreover, ERBB4 acquires 5 different mutations that coincide with its interfaces. These mutations are observed in cancer patients (derived from COSMIC). Genetic variations p. L39F, p.A90T, p. 409L and p.V468F mutations are coinciding with ERBB4-EREG interactions. Furthermore p. L39F and p.N352S mutations are coinciding with ERBB4-HBEGF interaction. Additionally, 4 mutations of EGFR derived from COSMIC database are coinciding with its interactions. While p. D46N, p.Q432H and p.V441I are affecting EGFR-EREG interaction, p. R377S mutation is affecting both EGFR-EREG and EGFR-HBEGF interactions.

These mutations may be making the mentioned interactions stronger or weaker but they are most probably changing the functions of the EREG, HBEGF, EGFR and ERBB4 proteins (Figure **6**). Besides, there may be a relationship between the metastasis progression and these mutations. 

### ELANE (ELA2)

ELANE interacts with CSF3 in BMSN, while it is switching its interaction partner to VCAM1 in LMSN. CSF3 is a seed gene in BMSN [13], while VCAM1 is a seed gene in LMSN [4]. The structural models of these interactions are not available in PDB, but we have PRISM predictions for these complexes. 

**Figure 7:**
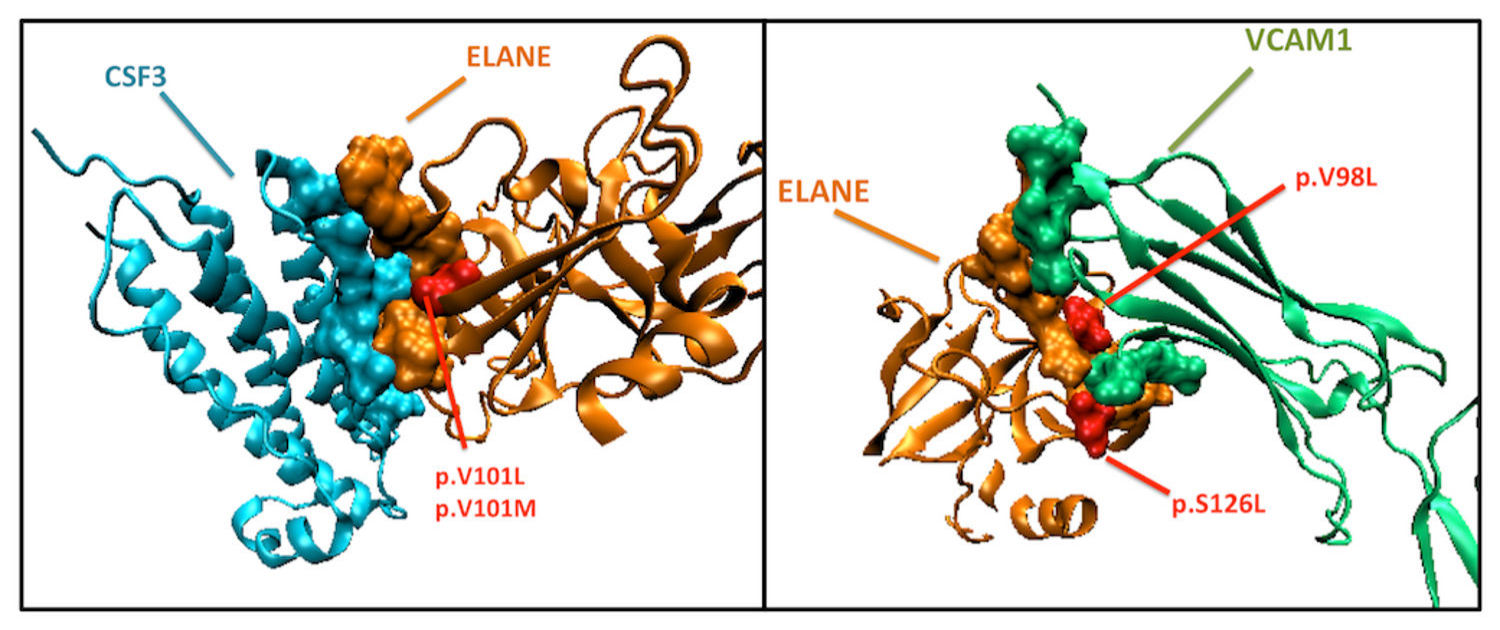
The PRISM predictions for ELANE (orange) - VCAM1 (green) and ELANE - CSF3 (blue) interaction. The amino acids 98, 101, 126 (red amino acids) on ELANE have genetic variations. Amino acid 101 is a hotspot in the CSF3 – ELANE interface, moreover amino acids 98 and 126 are part of the ELANE – VCAM1 interface.

ELANE has a variants that coincides with its interfaces (p. V98L, p.V101L, p.V101M and p. S126L (derived from UNIPROT)). The variances in the amino acid 101, which are polymorphisms, coincides with one of the hotspots of the interface region between ELANE and CSF3 and the variances in the amino acids 98 (polymorphism) and 126 (unclassified variation) are inside the interface region of VCAM1 on ELANE (Figure **7**). These amino-acid variances may be affecting the interactions of ELANE with CSF3 and VCAM1. As a result, these amino acid variations may be related with metastasis progression in breast cancer patients. 

## Conclusions

In this study we combined PPI networks, protein-protein interface structure and genetic variations together at the systems level to explain genotype-phenotype relationships. We have built two networks of proteins playing roles in different breast cancer metastasis and tried to explain the mechanisms behind metastasis process.

We built a comprehensive human PPI network, by combining the available PPI data from various databases. Then we ranked all the interactions of this network according to their relevance to genes that are known to be mediating breast cancer to brain and lung metastasis. Subsequently, we formed two distinct metastasis PPI sub-networks from high ranked interactions. Next, we introduced structural knowledge to metastasis PPI sub-networks. Only a small proportion of our protein complexes were available in PDB. We modeled the interface structures of PPIs by using PRISM tool. Knowing the interface structure between two proteins and the residue numbers on the interface surface, allowed us checking whether the mutations are located in the interfaces or not. 

We preformed functional analysis on metastasis sub-networks and observed that the proteins engaged in LMSN are enriched in “Infectious Diseases”, “Cancer” and “Immune System” KEGG classes. This correlation pinpoints a relationship between pathogens, immune system and lung metastasis. This may be due to the fact that, brain is a better-protected area than the lung, due to the blood-brain barrier and being less exposed to outside world compared to lung. Besides, the protein complexes in LMSN utilize more interface templates found in PPIs in microbes than BMSN. This finding reinforces our conclusion about the relationship between lung metastasis progression and pathogens. Furthermore, we saw that in both metastasis sub-networks the proteins using microbial interface architectures are mostly related with cell adhesion. Cell adhesion is a very important mechanism for metastasis and our findings suggest that there may be some mechanistic commonalities, such as cell adhesion, between pathogens and metastatic cancer cells employed during cell invasion. Actually, most of these proteins have interactions with proteins of pathogens themselves. 

We provided structural predictions for the architecture of interfaces of interactions between EGFR-EREG, EGFR-HBEGF, ERBB4-EREG, ERBB4-HBEGF, ELANE-CSF3 and ELANE-VCAM1. 

Conclusively, we built two different breast cancer metastasis PPI sub-networks, and made use of protein structures to explain the phenotype-genotype relationships. These network models may provide a foundation for future studies and may also be helpful for finding escape pathways of breast cancer metastasis. 

## Supporting Information

File S1 **Supporting figures and tables.** Figure S1, Structural enrichment of PPI networks with protein-protein interface predictions. FN1 and LTBP1 are predicted to be interacting via 1ywkAC template. This template is the interaction between A and C chains PDB ID: 1ywk complex. Figure S2, The increase in the number of interactions, as the number of GUILD score gets smaller. Figure S3, The increase in the number of interactions, as the number of nodes gets bigger. Table S1, Host-pathogen knowledge on proteins that use pathogenic interface architectures in BMSN. Table S2, Host-pathogen knowledge on proteins that use pathogenic interface architectures in LMSN. Table S3, The evidence for the presence of the genes of LMSN in different databases. Table S4, The evidence for the presence of the genes of BMSN in different databases. Table S5, The KEGG pathways enriched (P<0.05) in BMSN with respect to ClueGO p-value. Table S6, The KEGG pathways enriched (P<0.05) in LMSN with respect to ClueGO p-value. Table S7, The frequency of interfaces in both metastasis networks. Table S8, Proteins in BMSN that have PRISM interface predictions. Table S9, Proteins in LMSN that have PRISM interface predictions. Table S10, Distribution of the residue numbers and the mutation numbers per protein. Table S11, The total residues numbers/genetic variations observed in different locations and the odds ratio, 95% confidence interval, and the P-value for a two tailed test that OR is different from 1.0. Table S12, Interface residues (Sequence IDs) of HBEGF-EGFR model. The binding site residues of HBEGF protein’s complexes available in PDB and the binding site residues of EGFR protein’s complexes available in PDB. The interface residues that are overlaping with available binding site residues are in italic, bold fonts. Table S13, Interface residues (Sequence IDs) of EREG-EGFR model. The binding site residues of EGFR protein’s complexes available in PDB. The interface residues that are overlaping with available binding site residues are in italic, bold fonts. Table S14, Interface residues (Sequence IDs) of HBEGF-ERBB4 model. The binding site residues of HBEGF protein’s complexes available in PDB and the binding site residues of ERBB4 protein’s complexes available in PDB. The interface residues that are overlaping with available binding site residues are in italic, bold fonts. Table S15, Interface residues (Sequence IDs) of EREG-ERBB4 model. the binding site residues of ERBB4 protein’s complexes available in PDB. The interface residues that are overlaping with available binding site residues are in italic, bold fonts. Table S16, The interfaces in the 1jogCD PRINT cluster. Table S17, The biological processes of the proteins utilizing the most frequent interfaces of LMSN. Table S18, The molecular functions of the proteins utilizing the most frequent interfaces of LMSN. Table S19, The biological processes of the proteins utilizing the most frequent interfaces of BMSN. Table S20, The molecular functions of the proteins utilizing the most frequent interfaces of BMSN.
(DOC)
