# Supplementary material for: Integrating Structure to Protein-Protein Interaction Networks That Drive Metastasis to Brain and Lung in Breast Cancer
Source: PLoS One. 2013 Nov 22;8(11):e81035. doi: 10.1371/journal.pone.0081035 (PMC3838352; doi:10.1371/journal.pone.0081035)
Supplement: File S1 — Supporting figures and tables. Figure S1, Structural enrichment of PPI networks with protein-protein interface predictions. FN1 and LTBP1 are predicted to be interacting via 1ywkAC template. This template is the interaction between A and C chains PDB ID: 1ywk complex. Figure S2, The increase in the number of interactions, as the number of GUILD score gets smaller. Figure S3, The increase in the number of interactions, as the number of nodes gets bigger. Table S1, Host-pathogen knowledge on proteins that use pathogenic interface architectures in BMSN. Table S2, Host-pathogen knowledge on proteins that use pathogenic interface architectures in LMSN. Table S3, The evidence for the presence of the genes of LMSN in different databases. Table S4, The evidence for the presence of the genes of BMSN in different databases. Table S5, The KEGG pathways enriched (P<0.05) in BMSN with respect to ClueGO p-value. Table S6, The KEGG pathways enriched (P<0.05) in LMSN with respect to ClueGO p-value. Table S7, The frequency of interfaces in both metastasis networks. Table S8, Proteins in BMSN that have PRISM interface predictions. Table S9, Proteins in LMSN that have PRISM interface predictions. Table S10, Distribution of the residue numbers and the mutation numbers per protein. Table S11, The total residues numbers/genetic variations observed in different locations and the odds ratio, 95% confidence interval, and the P-value for a two tailed test that OR is different from 1.0. Table S12, Interface residues (Sequence IDs) of HBEGF-EGFR model. The binding site residues of HBEGF protein’s complexes available in PDB and the binding site residues of EGFR protein’s complexes available in PDB. The interface residues that are overlaping with available binding site residues are in italic, bold fonts. Table S13, Interface residues (Sequence IDs) of EREG-EGFR model. The binding site residues of EGFR protein’s complexes available in PDB. The interface residues that are overlaping with available [file pone.0081035.s001.doc]

**FIGURES:**


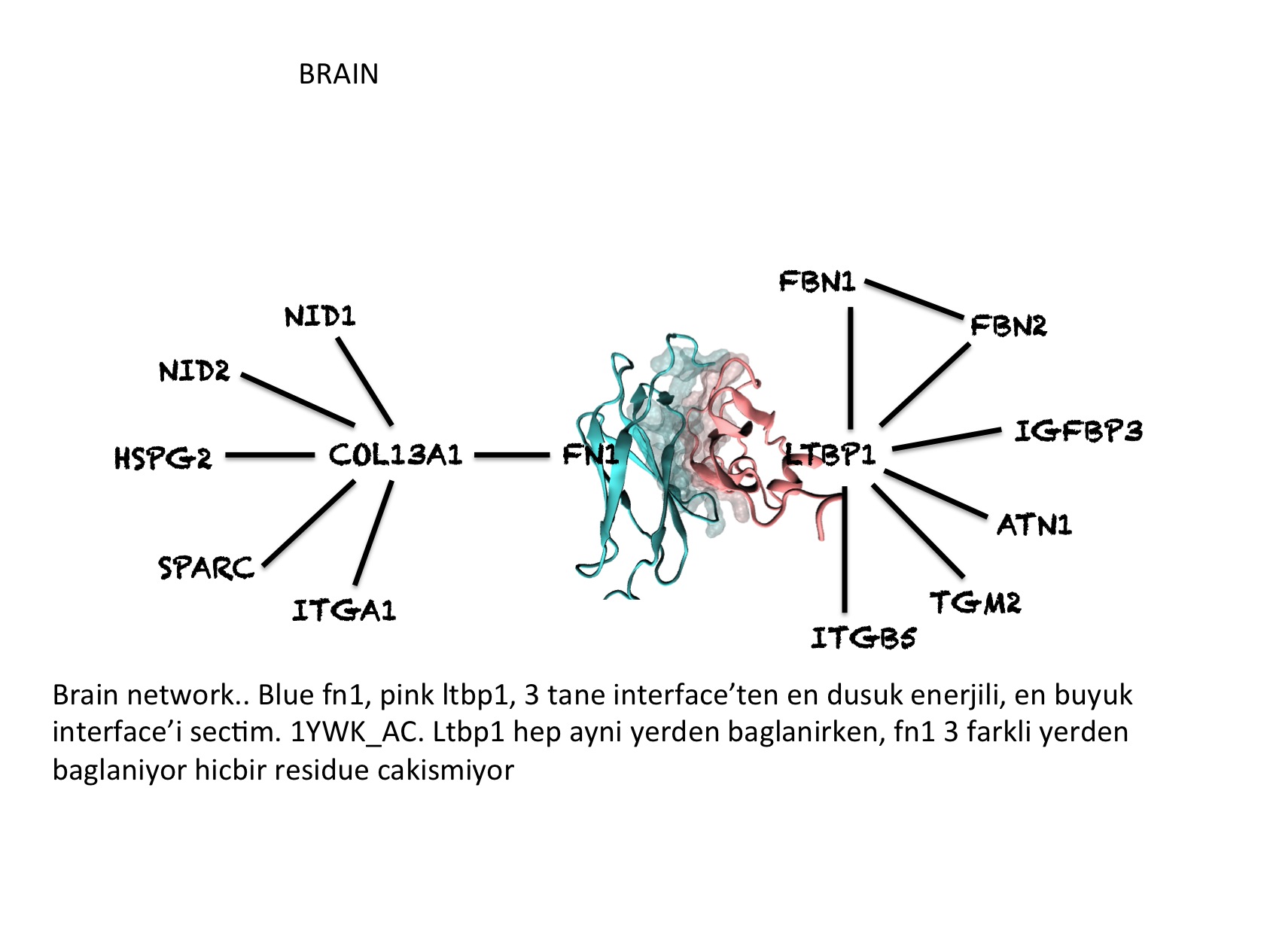


**Figure S1.** **Structural enrichment of PPI networks with protein-protein interface predictions.** FN1 and LTBP1 are predicted to be interacting via 1ywkAC template. This template is the interaction between A and C chains PDB ID: 1ywk complex.

**
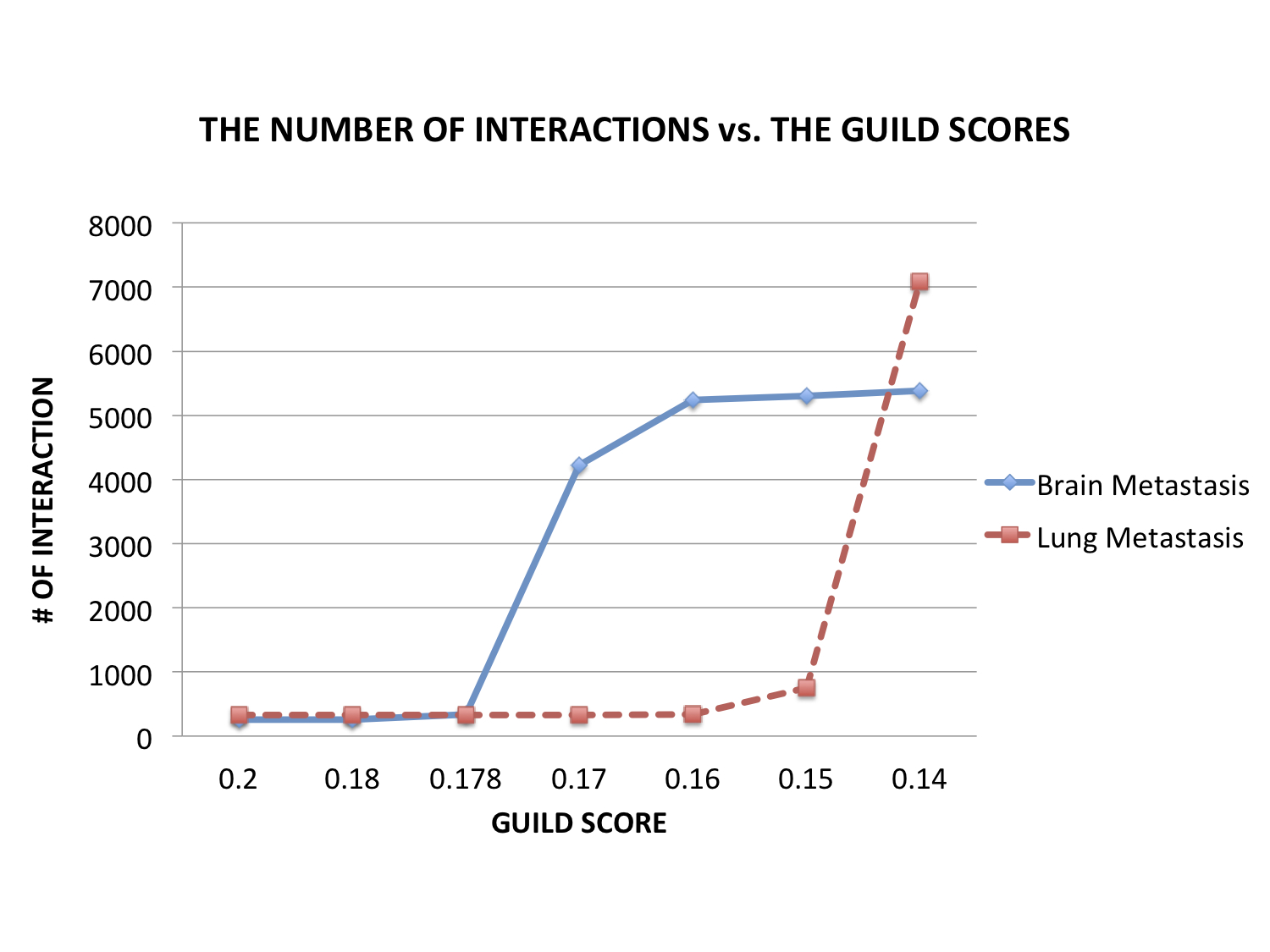
**

**Figure S2.** **The increase in the number of interactions, as the number of GUILD score gets smaller.**


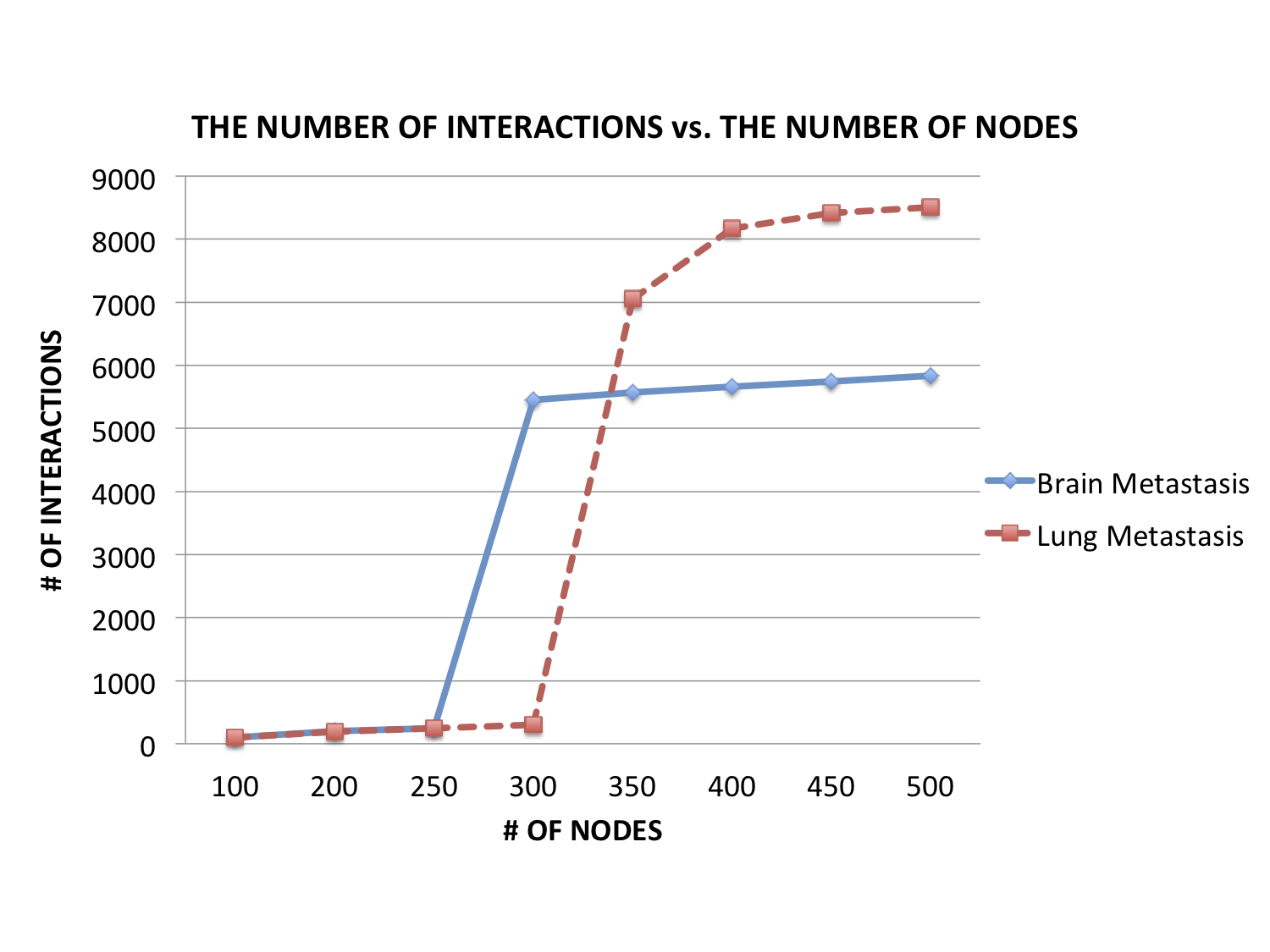


**Figure S3. The increase in the number of interactions, as the number of nodes gets bigger.**

**TABLES:**

**Table S1. Host-pathogen knowledge on proteins that use pathogenic interface architectures in BMSN.**

| **PROTEIN** | **RELATIONSHIP** | **SOURCE** |
| --- | --- | --- |
| LTBP1 | - | - |
| TNFSF11 | - | - |
| SERPINA3 | - | - |
| BSG | - | - |
| FBN1 | - | - |
| PLS3 | - | - |
| FSCN1 | - | - |
| FN1 | interaction with bacteria | [[1](#_ENREF_1)] |
| CCL2 | involvement in mycobacterium tuberculosis susceptibility | [[2](#_ENREF_2)] |
| MMP1 | Host-virus interaction | UNIPROT |
| ITGB1 | Host-virus interaction | UNIPROT |
| KPNB1 | Host-virus interaction | UNIPROT |
| CD44 | HIV1 downregulates | HPIDB |
| ITGA5 | Host-virus interaction | UNIPROT |

**Table S2. Host-pathogen knowledge on proteins that use pathogenic interface architectures in LMSN**.

| **PROTEIN** | **RELATIONSHIP** | **SOURCE** |
| --- | --- | --- |
| ABL1 | HIV1 downregulates | HPIDB |
| BCAR1 | - | - |
| BSG | - | - |
| CCL2 | involvement in mycobacterium tuberculosis susceptibility | [[2](#_ENREF_2)] |
| CD44 | HIV1 downregulates | HPIDB |
| CDH1 | - | - |
| CNTN1 | - | - |
| CRKL | - | - |
| CXCL1 | - | - |
| CXCR4 | Host-virus interaction | UNIPROT |
| ELA2 (elane) | associated with Hendra virus | HPIDB |
| EZR | - |  |
| FN1 | interaction with bacteria | [[1](#_ENREF_1)] |
| FSCN1 | - |  |
| IL13 | - |  |
| ITCH | Host-virus interaction | UNIPROT |
| ITGB1 | Host-virus interaction | UNIPROT |
| ITGB7 | Host cell receptor for virus entry | UNIPROT |
| JAK3 | - |  |
| KPNB1 | Host-virus interaction | UNIPROT |
| LTBP1 | - |  |
| MICAL1 | - |  |
| MMP1 | Host-virus interaction | UNIPROT |
| MMP9 | - |  |
| MYH9 | - |  |
| NEDD9 | - |  |
| PIK3CA | associated with influenza A virus | HPIDB |
| PLS3 | - |  |
| PTK2 | - |  |
| PTK2B | - |  |
| PTPN11 | - |  |
| PTPN6 | - |  |
| PTPRC | defense response to virus | UNIPROT |
| PXN | - |  |
| SERPINA3 | - |  |
| SMAD1 | - |  |
| TNC | - |  |
| TNFSF11 | - |  |
| VAV1 | - |  |
| VCAM1 | Host-virus interaction | UNIPROT |

**Table S3**. **The evidence for the presence of the genes of LMSN in different databases.**

**Table S4**. **The evidence for the presence of the genes of BMSN in different databases**

**Table S5. The KEGG pathways enriched (P<0.05) in BMSN with respect to ClueGO p-value.**

| **PATHWAY NAME** | **ClueGo PValue (after Bonferroni Correction)** | **KEGG Class** |
| --- | --- | --- |
| path:hsa04142 Lysosome | 5.47E-14 | Cellular Processes; Transport and Catabolism |
| path:hsa05222 Small cell lung cancer | 4.86E-06 | Human Diseases; Cancers |
| path:hsa04210 Apoptosis | 3.50E-05 | Cellular Processes; Cell Growth and Death |
| path:hsa03430 Mismatch repair | 0.001638707 | Genetic Information Processing; Replication and repair |
| path:hsa04145 Phagosome | 2.47E-04 | Cellular Processes; Transport and Catabolism |
| path:hsa04640 Hematopoietic cell lineage | 0.001806729 | Organismal Systems; Immune System |
| path:hsa04960 Aldosterone-regulated sodium reabsorption | 0.042397972 | Organismal Systems; Excretory system |
| path:hsa05146 Amoebiasis | 0.009476797 | Human Diseases; Infectious Diseases |
| path:hsa03460 Fanconi anemia pathway | 0.027029128 | Genetic Information Processing; Replication and repair |

**Table S6. The KEGG pathways enriched (P<0.05) in LMSN** with respect to ClueGO p-value.

| **PATHWAY NAME** | **ClueGo PValue (after Bonferroni Correction)** | **KEGG Class** |
| --- | --- | --- |
| path:hsa04062 Chemokine signaling pathway | 8.53E-16 | Organismal Systems; Immune System |
| path:hsa03010 Ribosome | 1.41E-08 | Genetic Information Processing; Translation |
| path:hsa04670 Leukocyte transendothelial migration | 1.65E-07 | Organismal Systems; Immune System |
| path:hsa05100 Bacterial invasion of epithelial cells | 7.15E-06 | Human Diseases; Infectious diseases |
| path:hsa04512 ECM-receptor interaction | 8.72E-05 | Environmental Information Processing; Signaling Molecules and Interaction |
| path:hsa04012 ErbB signaling pathway | 1.16E-04 | Environmental Information Processing; Signal transduction |
| path:hsa04722 Neurotrophin signaling pathway | 1.20E-04 | Organismal Systems; Nervous system |
| path:hsa04530 Tight junction | 8.94E-04 | Cellular Processes; Cell Communication |
| path:hsa04520 Adherens junction | 0.00293406 | Cellular Processes; Cell communication |
| path:hsa05142 Chagas disease (American trypanosomiasis) | 0.004287851 | Human Diseases; Infectious diseases |
| path:hsa05160 Hepatitis C | 0.004273745 | Human Diseases; Infectious Diseases |
| path:hsa05212 Pancreatic cancer | 0.010078577 | Human Diseases; Cancers |
| path:hsa04660 T cell receptor signaling pathway | 0.006243754 | Organismal Systems; Immune system |
| path:hsa05162 Measles | 0.004967513 | Human Diseases; Infectious Diseases |
| path:hsa05131 Shigellosis | 0.016765074 | Human Diseases; Infectious diseases |
| path:hsa05220 Chronic myeloid leukemia | 0.014252803 | Human Diseases; Cancers |
| path:hsa04910 Insulin signaling pathway | 0.020275511 | Organismal Systems; Endocrine System |
| path:hsa05213 Endometrial cancer | 0.027536913 | Human Diseases; Cancers |
| path:hsa05120 Epithelial cell signaling in Helicobacter pylori infection | 0.0372481 | Human Diseases; Infectious diseases |
| path:hsa04350 TGF-beta signaling pathway | 0.042778509 | Environmental Information Processing; Signal transduction |
| path:hsa04720 Long-term potentiation | 0.044536399 | Organismal Systems; Nervous system |

**Table S7.** The frequency of interfaces in both metastasis networks.

| Interface Template Name | Frequency in Lung Metastasis Network | Frequency in Brain Metastasis Network |  | Interface Template Name | Frequency in Lung Metastasis Network | Frequency in Brain Metastasis Network |
| --- | --- | --- | --- | --- | --- | --- |
| 2b8nAB | 8 | 2 |  | 1xedAC | 1 | 0 |
| 2a6aAB | 4 | 0 |  | 1l1yAD | 1 | 0 |
| 1nqlAB | 2 | 2 |  | 1on2AB | 1 | 0 |
| 1qjcAB | 2 | 2 |  | 1e8oCD | 1 | 0 |
| 1moxAC | 2 | 2 |  | 1a49AB | 1 | 0 |
| 1jogCD | 5 | 1 |  | 1iieAB | 1 | 0 |
| 1gveAB | 3 | 1 |  | 1fr3AB | 1 | 0 |
| 1oh0AB | 1 | 1 |  | 1djrDE | 1 | 0 |
| 1bqqMT | 1 | 1 |  | 1p65AB | 1 | 0 |
| 1zdnAB | 2 | 1 |  | 1symAB | 1 | 0 |
| 2bo4CD | 2 | 1 |  | 1c4zAD | 1 | 0 |
| 1tueAH | 1 | 1 |  | 1y0eAB | 1 | 0 |
| 1xx9CD | 1 | 1 |  | 1x8dAB | 1 | 0 |
| 1g8tAB | 1 | 1 |  | 1p5qAC | 1 | 0 |
| 1b3dAB | 1 | 1 |  | 1u6iAF | 1 | 0 |
| 1jflAB | 0 | 1 |  | 1u0kAB | 1 | 0 |
| 1okjAB | 0 | 1 |  | 1wb1BD | 1 | 0 |
| 1qiaCD | 1 | 1 |  | 1twjCD | 1 | 0 |
| 1eq2GJ | 0 | 1 |  | 1xqcAB | 1 | 0 |
| 2b99CE | 0 | 1 |  | 1tljAB | 1 | 0 |
| 1kkmAB | 1 | 1 |  | 1f6fBC | 1 | 0 |
| 2btfAP | 1 | 1 |  | 1p60AB | 1 | 0 |
| 1jyaAB | 1 | 1 |  | 1pbiAB | 1 | 0 |
| 1cd9AB | 0 | 1 |  | 1yw0AD | 1 | 0 |
| 1jzmAB | 1 | 1 |  | 1iawAB | 1 | 0 |
| 1nh0AB | 2 | 1 |  | 1v8pEF | 1 | 0 |
| 1rd5AB | 0 | 1 |  | 1qorAB | 1 | 0 |
| 1kamAB | 0 | 1 |  | 1rkeAB | 1 | 0 |
| 1ywkAC | 1 | 1 |  | 1o60AB | 1 | 0 |
| 1t6uAF | 2 | 0 |  | 1jd1AB | 1 | 0 |
| 1y9iAD | 2 | 0 |  | 1vr0BC | 1 | 0 |
| 1pe0AB | 1 | 0 |  | 1n1bAB | 1 | 0 |
| 1j2rCD | 1 | 0 |  | 1fiuAB | 1 | 0 |
| 1u2eAC | 1 | 0 |  | 3ezeAB | 1 | 0 |
| 1wmhAB | 1 | 0 |  | 1k2fAB | 1 | 0 |
| 1tb3AD | 1 | 0 |  | 1um0CD | 1 | 0 |
| 1sj1AB | 1 | 0 |  | 1vi6AB | 1 | 0 |
| 1zuwAC | 1 | 0 |  | 1q5cAB | 1 | 0 |
| 1xmzAB | 1 | 0 |  | 1s96AB | 1 | 0 |
| 2erbAB | 1 | 0 |  | 1yllAB | 1 | 0 |
| 1mzhAB | 1 | 0 |  | 1t3uAB | 1 | 0 |

**Table S8. Proteins in BMSN that have** PRISM interface predictions

| **PROTEIN** | **BIOLOGICAL FUNCTION** | **USING PATHOGEN INTERFACE ARCHITECTURE** |
| --- | --- | --- |
| LTBP1 | - | YES |
| SERPINA3 | - | YES |
| BSG | - | YES |
| FBN1 | - | YES |
| PLS3 | - | YES |
| FSCN1 | - | YES |
| MMP1 | - | YES |
| KPNB1 | - | YES |
| FN1 | cell adhesion | YES |
| CCL2 | cell adhesion | YES |
| ITGB1 | cell adhesion | YES |
| CD44 | cell adhesion | YES |
| ITGA5 | cell adhesion | YES |
| TNFSF11 | positive regulation of homotypic cell-cell adhesion | YES |
| CSF3R | cell adhesion | NO |
| HBEGF | - | NO |
| EGFR | cell-cell adhesion | NO |
| ELANE | - | NO |
| ERBB4 | - | NO |
| MMP7 | - | NO |
| TIMP1 | - | NO |

**Table S9.** **Proteins in LMSN that have PRISM interface predictions**

| **PROTEIN** | **BIOLOGICAL FUNCTION** | **USING PATHOGEN INTERFACE ARCHITECTURE** |
| --- | --- | --- |
| CDH1 | cell adhesion | YES |
| CNTN1 | cell adhesion | YES |
| TNC | cell adhesion | YES |
| BCAR1 | cell adhesion | YES |
| NEDD9 | cell adhesion | YES |
| PTK2B | cell adhesion | YES |
| PXN | cell adhesion | YES |
| ABL1 | cell adhesion | YES |
| FN1 | cell adhesion | YES |
| CCL2 | cell adhesion | YES |
| ITGB7 | cell adhesion | YES |
| ITGB1 | cell adhesion | YES |
| CD44 | cell adhesion | YES |
| ITGA5 | cell adhesion | YES |
| VCAM1 | cell adhesion | YES |
| MYH9 | cell-cell adhesion | YES |
| EZR | leukocyte cell-cell adhesion | YES |
| PTPRC | negative regulation of cell adhesion involved in substrate-bound cell migration | YES |
| PTK2 | negative regulation of cell-cell adhesion, positive regulation of cell adhesion | YES |
| TNFSF11 | positive regulation of homotypic cell-cell adhesion | YES |
| PTPN11 | regulation of cell adhesion mediated by integrin | YES |
| BSG | - | YES |
| CRKL | - | YES |
| CXCL1 | - | YES |
| IL13 | - | YES |
| JAK3 | - | YES |
| MMP9 | - | YES |
| SMAD1 | - | YES |
| FSCN1 | - | YES |
| LTBP1 | - | YES |
| MICAL1 | - | YES |
| PLS3 | - | YES |
| PTPN6 | - | YES |
| SERPINA3 | - | YES |
| VAV1 | - | YES |
| ELA2 (elane) | - | YES |
| MMP1 | - | YES |
| KPNB1 | - | YES |
| CXCR4 | - | YES |
| ITCH | - | YES |
| PIK3CA | - | YES |
| CD74 | - | NO |
| CHAT | - | NO |
| CRK | cell adhesion | NO |
| EGFR | cell-cell adhesion | NO |
| ERBB4 | - | NO |
| EREG | - | NO |
| SMAD3 | - | NO |
| MMP7 | - | NO |
| MSN | leukocyte cell-cell adhesion | NO |
| STAT1 | - | NO |
| TCF3 | - | NO |
| TIMP1 | - | NO |
| BCAR3 | - | NO |
| DIMT1L | - | NO |

**Table S10. Distribution of the residue numbers and the mutation numbers per protein.**

**
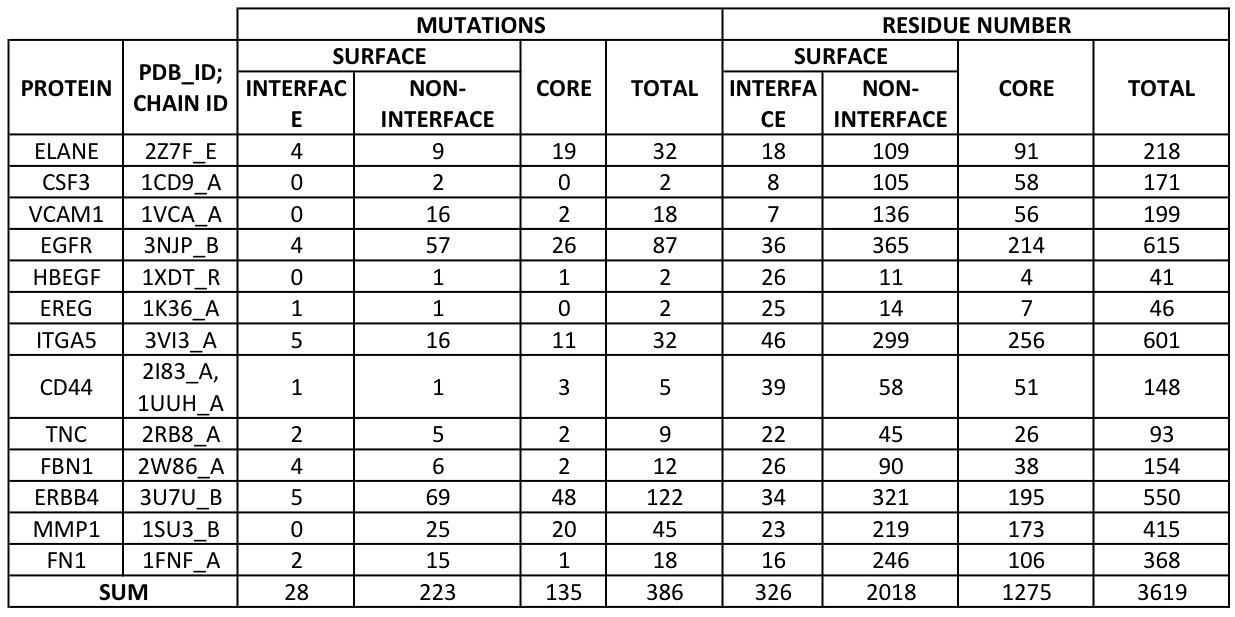
**

**Table S11. The total residues numbers/genetic variations observed in different locations and the odds ratio, 95% confidence interval, and the P-value for a two tailed test that OR is different from 1.0.**

|  | # of Residues | Genetic Variations |  | OR | 95 percent CI | P value |
| --- | --- | --- | --- | --- | --- | --- |
| Core | 1275 | 135 | Core vs. surface | 0.99 | 0.78 - 1.24 | 0.95 |
| Surface | 2344 | 251 | Interface vs surface noninterface | 0.76 | 0480 - 1.15 | 0.21 |
| Interface | 326 | 28 | Core vs. interface | 1.26 | 0.81 - 2.01 | 0.3 |
| Surface noninterface | 2018 | 223 |  |  |  |  |
| Total | 3619 | 386 |  |  |  |  |

**Table S12. Interface residues (Sequence IDs) of HBEGF-EGFR model. The binding site residues of HBEGF protein’s complexes available in PDB and the binding site residues of EGFR protein’s complexes available in PDB. The interface residues that are overlaping with available binding site residues are in italic, bold fonts.**

|  | **IN PDB** |  | **PRISM MODEL** | |  | **IN PDB** | | | |
| --- | --- | --- | --- | --- | --- | --- | --- | --- | --- |
| **PDB ID; Chain ID** | **1XDT ; R** |  | **-** | |  | **1NQL; A** | **1MOX; A** | **1IVO; A** | **3NJP; A** |
| **PROTEIN NAME** | **HBEGF** |  | **HBEGF** | **EGFR** |  | **EGFR** | **EGFR** | **EGFR** | **EGFR** |
| **RESIDUES** | 112 |  | 111 | ***36*** |  | 36 | 36 | 36 | 36 |
|  | 115 |  | ***112*** | ***37*** |  | 37 | 37 | 37 | 37 |
|  | 122 |  | 113 | ***38*** |  | 38 | 38 | 38 | 38 |
|  | 124 |  | 114 | ***39*** |  | 39 | 39 | 39 | 39 |
|  | 126 |  | ***115*** | ***40*** |  | 40 | 40 | 40 | 40 |
|  | 127 |  | 117 | ***41*** |  | 41 | 41 | 41 | 41 |
|  | 129 |  | ***124*** | ***42*** |  | 42 | 42 | 42 | 42 |
|  | 130 |  | ***126*** | ***69*** |  | 46 | 69 | 46 | 46 |
|  | 131 |  | ***127*** | ***92*** |  | 47 | 93 | 50 | 53 |
|  | 132 |  | ***129*** | ***93*** |  | 93 | 122 | 69 | 69 |
|  | 133 |  | ***131*** | ***122*** |  | 114 | 123 | 92 | 93 |
|  | 134 |  | ***132*** | ***123*** |  | 122 | 125 | 93 | 114 |
|  | 135 |  | 133 | ***125*** |  |  | 126 | 114 | 122 |
|  | 136 |  | ***134*** | ***349*** |  |  | 149 | 122 | 123 |
|  | 137 |  | ***139*** | ***377*** |  |  | 152 | 123 | 125 |
|  | 138 |  | ***140*** | ***379*** |  |  | 349 | 125 | 349 |
|  | 139 |  | ***141*** | ***380*** |  |  | 370 | 349 | 370 |
|  | 140 |  | 142 | ***381*** |  |  | 372 | 370 | 372 |
|  | 141 |  | ***143*** | ***408*** |  |  | 374 | 372 | 373 |
|  | 143 |  | 144 | ***433*** |  |  | 378 | 372 | 374 |
|  | 147 |  | 145 |  |  |  | 379 | 374 | 377 |
|  |  |  |  |  |  |  | 380 | 375 | 379 |
|  |  |  |  |  |  |  | 381 | 381 | 380 |
|  |  |  |  |  |  |  | 406 | 382 | 381 |
|  |  |  |  |  |  |  | 408 | 406 | 382 |
|  |  |  |  |  |  |  | 432 | 408 | 406 |
|  |  |  |  |  |  |  | 433 | 432 | 408 |
|  |  |  |  |  |  |  | 435 | 433 | 432 |
|  |  |  |  |  |  |  | 436 | 435 | 433 |
|  |  |  |  |  |  |  | 439 | 436 | 436 |
|  |  |  |  |  |  |  | 462 | 439 | 439 |
|  |  |  |  |  |  |  |  | 441 | 441 |
|  |  |  |  |  |  |  |  | 462 | 462 |
|  |  |  |  |  |  |  |  | 464 | 464 |
|  |  |  |  |  |  |  |  | 489 | 491 |
|  |  |  |  |  |  |  |  |  | 492 |

**Table S13. Interface residues (Sequence IDs) of EREG-EGFR model. The binding site residues of EGFR protein’s complexes available in PDB. The interface residues that are overlaping with available binding site residues are in italic, bold fonts.**

|  | **PRISM MODEL** | | |  | **IN PDB** | | | |
| --- | --- | --- | --- | --- | --- | --- | --- | --- |
| **PDB ID; Chain ID** | **-** | | |  | **1NQL; A** | **1MOX; A** | **1IVO; A** | **3NJP; A** |
| **PROTEIN NAME** | **EREG** | | **EGFR** |  | **EGFR** | **EGFR** | **EGFR** | **EGFR** |
| **RESIDUES** | 71 | | 34 |  | 36 | 36 | 36 | 36 |
|  | 72 | | 35 |  | 37 | 37 | 37 | 37 |
|  | 73 | | ***36*** |  | 38 | 38 | 38 | 38 |
|  | 74 | | ***38*** |  | 39 | 39 | 39 | 39 |
|  | 75 | | ***39*** |  | 40 | 40 | 40 | 40 |
|  | 77 | | ***40*** |  | 41 | 41 | 41 | 41 |
|  | 84 | | ***41*** |  | 42 | 42 | 42 | 42 |
|  | 86 | | ***42*** |  | 46 | 69 | 46 | 46 |
|  | 87 | | 43 |  | 47 | 93 | 50 | 53 |
|  | 89 | | ***46*** |  | 93 | 122 | 69 | 69 |
|  | 91 | | ***69*** |  | 114 | 123 | 92 | 93 |
|  | 92 | | ***93*** |  | 122 | 125 | 93 | 114 |
|  | 93 | | ***122*** |  |  | 126 | 114 | 122 |
|  | 94 | | ***123*** |  |  | 149 | 122 | 123 |
|  | ***96*** | **C-Terminus** | ***125*** |  |  | 152 | 123 | 125 |
|  | ***99*** |  | ***372*** |  |  | 349 | 125 | 349 |
|  | ***101*** |  | ***373*** |  |  | 370 | 349 | 370 |
|  | ***104*** |  | ***374*** |  |  | 372 | 370 | 372 |
|  | ***105*** |  | ***377*** |  |  | 374 | 372 | 373 |
|  | ***106*** |  | ***379*** |  |  | 378 | 372 | 374 |
|  | 107 | | ***380*** |  |  | 379 | 374 | 377 |
|  | 108 | | ***381*** |  |  | 380 | 375 | 379 |
|  |  | | ***382*** |  |  | 381 | 381 | 380 |
|  |  | | 383 |  |  | 406 | 382 | 381 |
|  |  | | ***406*** |  |  | 408 | 406 | 382 |
|  |  | | ***408*** |  |  | 432 | 408 | 406 |
|  |  | | ***432*** |  |  | 433 | 432 | 408 |
|  |  | | ***433*** |  |  | 435 | 433 | 432 |
|  |  | | ***439*** |  |  | 436 | 435 | 433 |
|  |  | | ***441*** |  |  | 439 | 436 | 436 |
|  |  | | 442 |  |  | 462 | 439 | 439 |
|  |  | | ***462*** |  |  |  | 441 | 441 |
|  |  | | ***489*** |  |  |  | 462 | 462 |
|  |  | |  |  |  |  | 464 | 464 |
|  |  | |  |  |  |  | 489 | 491 |
|  |  | |  |  |  |  |  | 492 |

**Table S14. Interface residues (Sequence IDs) of HBEGF-ERBB4 model. The binding site residues of HBEGF protein’s complexes available in PDB and the binding site residues of ERBB4 protein’s complexes available in PDB. The interface residues that are overlaping with available binding site residues are in italic, bold fonts.**

|  | **IN PDB** |  | **PRISM MODEL** | |  | **IN PDB** |
| --- | --- | --- | --- | --- | --- | --- |
| **PDB ID; Chain ID** | **1XDT ; R** |  | **-** | |  | **3U7U; A** |
| **PROTEIN NAME** | **HBEGF** |  | **HBEGF** | **ERBB4** |  | **ERBB4** |
| **RESIDUES** | 112 |  | 111 | 33 |  | 34 |
|  | 115 |  | ***112*** | ***34*** |  | 35 |
|  | 122 |  | 113 | ***35*** |  | 36 |
|  | 124 |  | 114 | 36 |  | 37 |
|  | 126 |  | ***115*** | ***37*** |  | 38 |
|  | 127 |  | 117 | ***38*** |  | 39 |
|  | 129 |  | 118 | ***39*** |  | 40 |
|  | 130 |  | ***124*** | ***40*** |  | 41 |
|  | 131 |  | ***126*** | 52 |  | 42 |
|  | 132 |  | ***127*** | ***91*** |  | 44 |
|  | 133 |  | ***131*** | ***120*** |  | 51 |
|  | 134 |  | ***132*** | 121 |  | 91 |
|  | 135 |  | ***133*** | ***125*** |  | 111 |
|  | 136 |  | ***134*** | ***352*** |  | 112 |
|  | 137 |  | ***135*** | ***375*** |  | 120 |
|  | 138 |  | ***138*** | ***376*** |  | 121 |
|  | 139 |  | ***139*** | ***377*** |  | 123 |
|  | 140 |  | ***140*** | ***382*** |  | 125 |
|  | 141 |  | ***141*** | ***383*** |  | 148 |
|  | 143 |  | 142 | ***384*** |  | 346 |
|  | 147 |  | 144 | ***411*** |  | 369 |
|  |  |  | 145 | ***435*** |  | 370 |
|  |  |  | 146 | ***443*** |  | 371 |
|  |  |  | ***147*** | ***444*** |  | 382 |
|  |  |  |  |  |  | 383 |
|  |  |  |  |  |  | 384 |
|  |  |  |  |  |  | 385 |
|  |  |  |  |  |  | 405 |
|  |  |  |  |  |  | 429 |
|  |  |  |  |  |  | 432 |
|  |  |  |  |  |  | 435 |
|  |  |  |  |  |  | 437 |
|  |  |  |  |  |  | 438 |
|  |  |  |  |  |  | 459 |

**Table S15. Interface residues (Sequence IDs) of EREG-ERBB4 model. the binding site residues of ERBB4 protein’s complexes available in PDB. The interface residues that are overlaping with available binding site residues are in italic, bold fonts.**

|  | **PRISM MODEL** | | |  | **IN PDB** |
| --- | --- | --- | --- | --- | --- |
| **PDB ID; Chain ID** | **-** | | |  | **3U7U; A** |
| **PROTEIN NAME** | **EREG** | | **ERBB4** |  | **ERBB4** |
| **RESIDUES** | 71 | | 32 |  | 34 |
|  | 72 | | 33 |  | 35 |
|  | 73 | | ***34*** |  | 36 |
|  | 74 | | ***36*** |  | 37 |
|  | 75 | | ***37*** |  | 38 |
|  | 77 | | ***38*** |  | 39 |
|  | 78 | | ***39*** |  | 40 |
|  | 84 | | ***40*** |  | 41 |
|  | 86 | | ***41*** |  | 42 |
|  | 87 | | ***44*** |  | 44 |
|  | 89 | | 52 |  | 51 |
|  | 91 | | 90 |  | 91 |
|  | 93 | | ***91*** |  | 111 |
|  | 94 | | ***120*** |  | 112 |
|  | 96 | **C-terminus** | ***121*** |  | 120 |
|  | 99 |  | 373 |  | 121 |
|  | 100 |  | ***375*** |  | 123 |
|  | 101 |  | ***376*** |  | 125 |
|  | 102 |  | ***377*** |  | 148 |
|  | 104 |  | ***383*** |  | 346 |
|  | 105 |  | 409 |  | 369 |
|  | 106 |  | ***411*** |  | 370 |
|  | 107 | | ***435*** |  | 371 |
|  | 108 | | ***443*** |  | 382 |
|  |  |  | ***444*** |  | 383 |
|  |  |  | ***465*** |  | 384 |
|  |  |  | 467 |  | 385 |
|  |  |  | 468 |  | 405 |
|  |  |  | 494 |  | 429 |
|  |  |  |  |  | 432 |
|  |  |  |  |  | 435 |
|  |  |  |  |  | 437 |
|  |  |  |  |  | 438 |
|  |  |  |  |  | 459 |

**Table S16. The interfaces in the 1jogCD PRINT cluster.**

| **PDB chains** | **Protein Name** | **Organism** | **Molecular Function** | **Biological Process** |
| --- | --- | --- | --- | --- |
| 1jog_CD/AB | uncharacterized protein | bacteria | - | - |
|
| 1i4y_AH/FG/BC/DE : 1i4z_CF/AD/BG/EH : 2hmqCD : 2hmzCD : 1hmdCD : 1hmoCD | Hemerythrin | eukaryota | - | Oxygen transport, Transport |
|
| 1wwpAB | uncharacterized protein | bacteria | - | - |
|
| 1wty_BC/AD | uncharacterized protein | bacteria | - | - |
|

**Table S17.** **The biological processes of the proteins utilizing the most frequent interfaces of LMSN**

**Table S18**. **The molecular functions of the proteins utilizing the most frequent interfaces of LMSN**

**Table S19**. **The biological processes of the proteins utilizing the most frequent interfaces of BMSN**.

**Table S20**. **The molecular functions of the proteins utilizing the most frequent interfaces of BMSN**

**REFERENCES**:

1. Schwarz-Linek U, Werner JM, Pickford AR, Gurusiddappa S, Kim JH, Pilka ES, Briggs JA, Gough TS, Hook M, Campbell ID, Potts JR: **Pathogenic bacteria attach to human fibronectin through a tandem beta-zipper.** *Nature* 2003, **423:**177-181.

2. Flores-Villanueva PO, Ruiz-Morales JA, Song CH, Flores LM, Jo EK, Montano M, Barnes PF, Selman M, Granados J: **A functional promoter polymorphism in monocyte chemoattractant protein-1 is associated with increased susceptibility to pulmonary tuberculosis.** *The Journal of experimental medicine* 2005, **202:**1649-1658.
